# Distinct Type of Transmission Barrier Revealed by Study of Multiple Prion Determinants of Rnq1

**DOI:** 10.1371/journal.pgen.1000824

**Published:** 2010-01-22

**Authors:** Michele L. Kadnar, Gulnara Articov, Irina L. Derkatch

**Affiliations:** Department of Microbiology, New York University School of Medicine, New York, New York, United States of America; Stanford University School of Medicine, United States of America

## Abstract

Prions are self-propagating protein conformations. Transmission of the prion state between non-identical proteins, e.g. between homologous proteins from different species, is frequently inefficient. Transmission barriers are attributed to sequence differences in prion proteins, but their underlying mechanisms are not clear. Here we use a yeast Rnq1/[*PIN^+^*]-based experimental system to explore the nature of transmission barriers. [*PIN^+^*], the prion form of Rnq1, is common in wild and laboratory yeast strains, where it facilitates the appearance of other prions. Rnq1's prion domain carries four discrete QN-rich regions. We start by showing that Rnq1 encompasses multiple prion determinants that can independently drive amyloid formation *in vitro* and transmit the [*PIN^+^*] prion state *in vivo*. Subsequent analysis of [*PIN^+^*] transmission between Rnq1 fragments with different sets of prion determinants established that (i) one common QN-rich region is required and usually sufficient for the transmission; (ii) despite identical sequences of the common QNs, such transmissions are impeded by barriers of different strength. Existence of transmission barriers in the absence of amino acid mismatches in transmitting regions indicates that in complex prion domains multiple prion determinants act cooperatively to attain the final prion conformation, and reveals transmission barriers determined by this cooperative fold.

## Author Summary

Prions, self-propagating protein conformations and causative agents of lethal neurodegenerative diseases, present a serious public health threat: they can arise sporadically and then spread by transmission to the same, as well as other, species. The risk of infecting humans with prions originating in wild and domestic animals is determined by the so-called transmission barriers. These barriers are attributed to differences in prion proteins from different species, but their underlying mechanisms are not clear. Recent findings that the prion state is transmitted through the interaction between short transmitting regions within prion domains revealed one type of transmission barrier, where productive templating is impeded by non-matching amino acids within transmitting regions. Here we present studies of the prion domain of the [*PIN^+^*]-forming protein, Rnq1, and describe a distinct type of transmission barrier not involving individual amino acid mismatches in the transmitting regions. Rnq1's prion domain is complex and encompasses four regions that can independently transmit the prion state. Our data suggest that multiple prion determinants of a complex prion domain act cooperatively to attain the prion conformation, and transmission barriers occur between protein variants that cannot form the same higher order structure, despite the identity of the region(s) driving the transmission.

## Introduction

An increasing number of proteins has been found to form β-sheet-rich aggregates called amyloid. In amyloid fibers, individual protein molecules are stacked on top of each other through the formation of inter-molecular β-strands perpendicular to the fiber axis. Existing fibers template the conformational conversion of the protein molecules with the same amino acid sequence, making amyloid an aggregate-based self-propagating protein conformation [1],[2].

Intracellular aggregates or extracellular amyloid deposits are hallmarks of over 30 hereditary and sporadic disorders including Creutzfeldt-Jacob, Alzheimer's, Parkinson's and Huntington's diseases and type II diabetes [2]. Several lines of evidence also suggest that the amyloid state has been functionally harnessed by organisms as diverse as bacteria, fungi and humans [3]–[5]. Discovery of infectivity of Creutzfeldt-Jacob disease and other spongiform encephalopathies, and linking the infectivity to the presence of the aggregated conformation of the PrP protein, PrP^Sc^, singled out these diseases as particularly hazardous, while PrP^Sc^ was termed “proteinaceous infectious agent”, or prion [6],[7]. However, recent studies that revealed the inherent transmissibility of several mammalian amyloidoses blurred the border between prions and other amyloids [8],[9].

In *Saccharomyces cerevisiae*, three epigenetic factors, [*PSI^+^*], [*URE3*] and [*PIN^+^*], manifest self-perpetuating amyloid conformations of Sup35, Ure2 and Rnq1, respectively [10]. The prion nature of these factors was established using genetic criteria proposed by Wickner [11], and the final proof of their protein-only transmission was obtained by infecting yeast cells with *in vitro*-made Sup35, Ure2 or Rnq1 amyloids [12]–[15]. Another recently discovered prion, [*SWI^+^*], satisfies the genetic criteria, but its amyloid nature has yet to be confirmed [16]. Yeast prions appear spontaneously or can be induced by transient overproduction of their respective prion-forming proteins [11], [16]–[19]. Once established, they are efficiently transmitted to daughter cells in mitosis and segregate in a non-Mendelian fashion in meiosis, and the [*PRION^+^*] state is maintained in the population until it is spontaneously lost or selectively eliminated. Phenotypes caused by the presence of [*PSI^+^*], [*URE3*] and [*SWI^+^*] are equivalent to loss-of-function mutations in, respectively, Sup35, Ure2 and Swi1, as these normally soluble proteins become sequestered into prion aggregates. For example, Sup35 is a translation termination factor, and [*PSI^+^*] increases the level of readthrough at stop codons [20], and can be detected as a suppressor of nonsense mutations [21]. Whether yeast prions are physiological epigentic modifiers of cellular functions, egoistic elements or diseases is a subject of debate [10], [22]–[30]. Nevertheless, yeast prions provide an excellent experimental model for addressing questions pertaining to self-propagating protein conformations.

The importance of direct templating in the transmission of the prion state is widely acknowledged, but the exact rules and determinants of this process remain unclear. Prion domains, which are terminally located in fungal prions, are essential and sufficient for prion formation and maintenance [18], [31]–[34]. A recent study implicates short sequences within prion domains as nucleation/recognition sites for prion formation [34]. In Sup35, which together with Ure2 and Rnq1 belongs to a class of proteins with QN-rich prion domains, recognition sites were mapped to two exceptionally QN-rich regions. A short peptide partially overlapping the primary N-terminal recognition site was previously shown to form in-register β-sheets stabilized by hydrogen bonds between Q and N residues [35].

Differences in amino acid sequences within prion domains can lead to transmission barriers, which make conversion of homologous proteins from different species impossible or inefficient [6]. For Sup35, transmission barriers were observed even between closely related species and between wild type and mutant alleles of the same protein, and were explained by amino acid mismatches within the N-terminal recognition site [34],[36],[37].

Yet, the prion phenomenon cannot be reduced to the stacking of short regions of the protein, as several lines of evidence underscore the importance of an overall conformation of the prion aggregate. For example, faithful propagation of [*PSI^+^*] involves parts of the Sup35 prion domain located outside of the presumptive recognition sites, e.g. a stretch of oligopeptide repeats [38]–[41]. Also, all prions exist as distinct heritable variants, called strains [18], [42]–[44]. Prion strains manifest distinct prion conformations of the same protein and, despite identical amino acid sequence, sometimes have different transmission barriers [45]–[47]. Both *in vitro* analysis and genetic data indicate that large regions of prion domains define strain differences [48]–[51].

[*PIN^+^*] stands out among yeast prions as it is found in industrial and pathogenic yeast isolates, while [*PSI^+^*] and [*URE3*] are common only in laboratory strains [24],[26]. Several [*PIN^+^*]-associated phenotypes indicate its engagement in a broad spectrum of interactions with other prions and amyloids [52]. As its name implies, [*PIN^+^*] (for [***P*** *SI^+^*]-**in**ducibility) is required for the *de novo* formation of [*PSI^+^*] [19],[53],[54]. It also facilitates the appearance of various QN-rich and non-QN-rich prions and polyQ aggregates [44],[55],[56]. Current evidence suggests that [*PIN^+^*] promotes prion formation through direct cross-seeding [57],[58]. On the other hand, co-existence of [*PIN^+^*] and other prions or aggregating proteins may result in prion loss and in toxicity of polyQ-encompassing proteins [59],[60]. The mechanisms of prion incompatibility are not clear, but several studies show involvement of chaperones and endosomes/cytoskeleton in [*PIN^+^*]-related polyQ toxicity [61]–[63]. Consistent with all [*PIN^+^*] phenotypes being a gain of function, the disruption of *RNQ1* does not make cells Pin^+^. On the contrary, *rnq1-Δ* interferes with increased frequency of spontaneous *de novo* appearance of [*PSI^+^*] in *ubc4* mutants [64]. Since, despite the long-term efforts of several labs, no biological function could be assigned to the non-aggregated conformation of Rnq1, there is a possibility that ability to aggregate or to interact with QN-rich or otherwise aggregation-prone proteins is key to its function.

**Figure 1:**
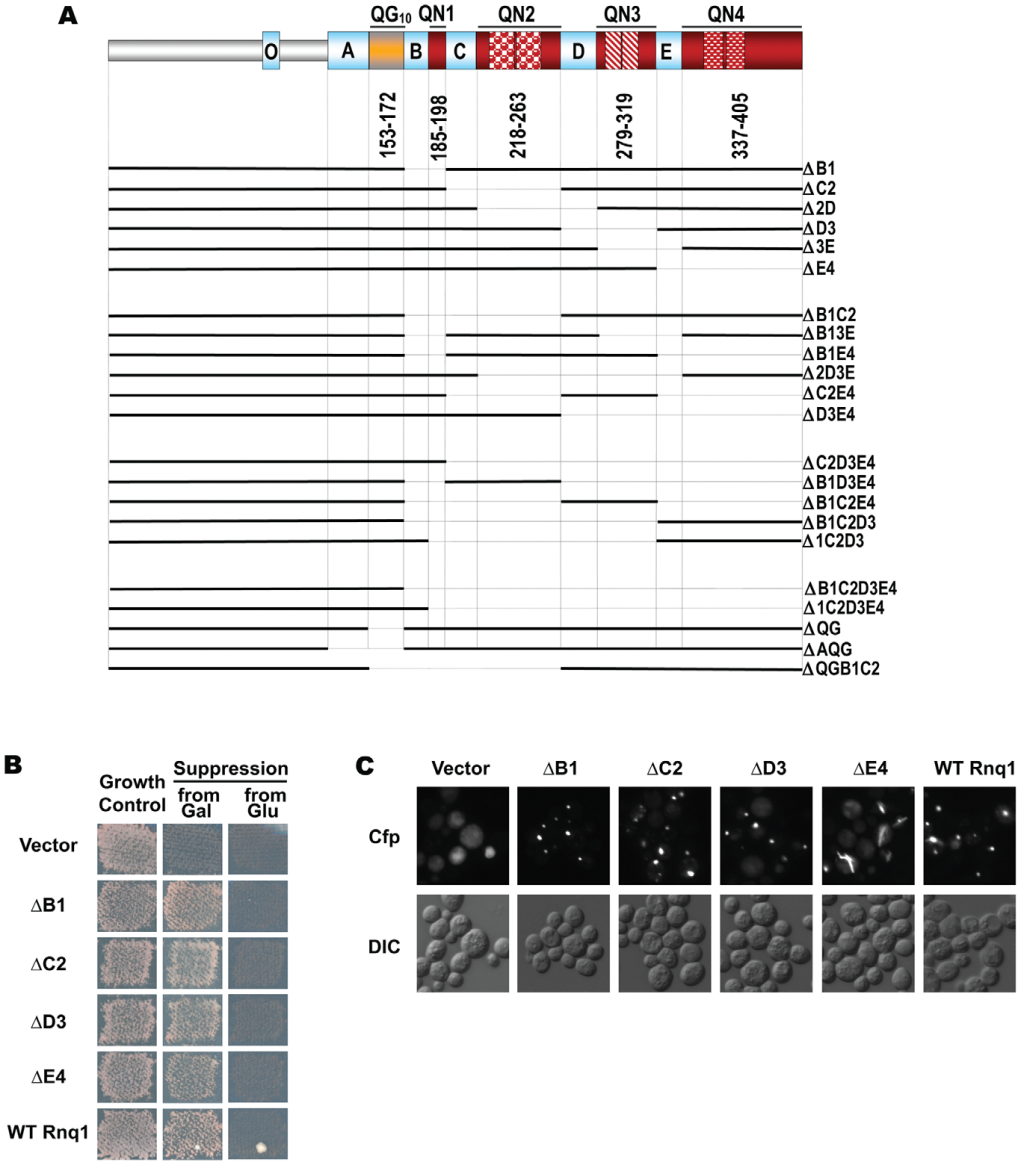
No one QN region of Rnq1 is essential for [*PIN^+^*] maintenance. (A) Schematic diagram of Rnq1 and deletion constructs. QN-rich regions are in red (patterned blocks indicate oligopeptide repeats), QG_10_, is in orange, hydrophobic patches are in blue. Numeric intervals indicate aa boundaries of respective QN regions. For deletion constructs, lines indicate regions present; nomenclature refers to deleted regions. (B) Deletion of any one QN region does not lead to the loss of ability to maintain [*PIN^+^*]. Indicated *LEU2*-marked constructs were transformed into [*PIN^+^*][*psi^−^*] *rnq1-Δ* 74-D694 carrying a *URA3*-marked *RNQ1* maintainer and a *HIS*-marked pGAL-SUP35NM::YFP [*PSI^+^*]-inducer. After selective elimination of the maintainer on FOA, expression of *SUP35NM::YFP* was transiently induced on SGal-Leu,His, and yeast were transferred to adeninelss media to score for [*PSI^+^*] (“from Gal”; shown is growth on SD-Ade after 10 days at 20°C) and to SD-Leu,His (“Growth Control”). In the control experiment yeast were grown on non-inducing SD-Leu,His instead of SGal-Leu,His (“from Glu”). (C) Rnq1 fragments introduced into the [*PIN^+^*] strain sustain the aggregated state after elimination of full-length Rnq1. Cultures carrying the indicated *LEU2*-marked plasmids were crossed to [*pin^−^*] 64-D697 carrying the *URA3*-marked pCUP-RNQ1::CFP. Diploids were selected on SD-Ura,Leu; reporter was induced by supplementing SD-Ura,Leu with 20µM CuSO_4_.

Lack of known function for soluble Rnq1 complicates defining the [*PIN^+^*] prion domain. The presumptive prion domain (aa 132–405 or 153–405) includes all Q- and QN-rich regions. *In vivo*, Rnq1_132-405_ can maintain [*PIN^+^*], and Rnq1_153-405_ can form a stable prion when fused to Sup35 lacking the [*PSI^+^*] prion domain [33],[65]. *In vitro*, these fragments form amyloid fibers, which can convert [*pin^−^*] cells into [*PIN^+^*] [15],[58],[66]. If these prion domain boundaries are correct, then the formation and maintenance of [*PIN^+^*] is driven by an extremely long and complex prion domain (Figure 1A; see below). This complexity has been noted previously [26],[65], however, no systematic analysis of the role of different structural determinants in [*PIN^+^*] formation and maintenance has been performed. Here we show that prion domain of Rnq1 carries multiple prion determinants that can independently maintain [*PIN^+^*]. Characterization of this complex prion domain allowed us to explore the role of individual prion determinants and overall prion fold in transmission of the prion state. We found that the ability to transmit the prion state is an intrinsic property of individual determinants/transmitting regions: one common region is required and generally sufficient for transmission of [*PIN^+^*] between Rnq1 fragments. However, efficiency of transmission between partially overlapping Rnq1 fragments is impeded by barriers due to differences in overall prion folds created by cooperative action of all prion determinants. This type of transmission barriers is clearly distinct from previously discussed barriers determined by aa mismatches in transmitting regions.

## Results

The C-terminal part of Rnq1 encompasses a stretch of ten QG two-residue repeats, QG_10_, and four QN-rich regions interspersed by hydrophobic sequences (Figure 1A). In this work, we refer to QN-rich regions as QN1 through QN4, and hydrophobic patches preceding QG_10_, QN1, QN2, QN3 and QN4 are labeled A, B, C, D and E, respectively. QN1 is short and simple, whereas other QNs each contain two imperfect repeats: a tandem 11 aa repeat in QN3 - **NQQQY^N^/_Q_QQGQN**, a 12 aa repeat separated by 2 aa in QN4 - **GQQQ^A^/_S_NEYGRPQ**, and a more degenerate 14 aa repeat separated by 1 aa in QN2 - **NS^Q^/-** **QGYN** **^−^/_N_** **^S^/_Q_YQ^N^/_G_GN**. Hydrophobic patches, for which helical structure is predicted (http://www.predictprotein.org; [67]), have a common **LA^S^/_A_** **^L^/_M_A** core sequence. More pair-wise similarity is seen for B and C - **SF^G^/_T_ALAS^L^/_M_** **ASSFM**, and D and E - **FS^S^/_A_LASMA^Q^/_S_SYLG**. Interestingly, a similar hydrophobic patch in the N-terminal part of Rnq1 (**LALLA**, aa 94–98), has recently been implicated in the interaction with the Sis1 chaperone ([68]; indicated as O in Figure 1A).

To assess the role of individual sequence determinants of Rnq1 in maintaining and transmitting the [*PIN^+^*] prion, we designed a series of deletions lacking various combinations of Q-rich regions and adjacent hydrophobic patches (Figure 1A). For all these constructs: (i) the non-QN-rich N-terminal domain preceding the A patch (aa 1–132) is retained; (ii) deletions start/end at the borders of QG_10_ or QN-rich regions; (iii) internal deletions are “seamless”, i.e. no additional amino acids are inserted at the junctions; (iv) no tags are attached at the termini to avoid possible interference of tag sequences; (v) in yeast, the expression of deletion constructs is controlled by the native *RNQ1* promoter, the native *RNQ1* terminator sequence follows the ORFs, and the constructs are introduced on a single-copy *CEN* vector.

### No one QN-rich region is essential for [*PIN* ^+^] maintenance

We first asked if any of the QN regions is essential for the maintenance of [*PIN^+^*]. Deletion constructs lacking one of the four QN regions, as well as an adjacent hydrophobic sequence (Figure 1A), were transformed into a [*PIN^+^*][*psi^−^*] *rnq1-Δ* 74-D694 strain expressing full-length *RNQ1* controlled by the *RNQ1* promoter. The plasmid-borne *RNQ1* maintainer kept steady-state Rnq1 levels comparable to those in strains with endogenous *RNQ1* and ensured stable maintenance of [*PIN^+^*] (not shown); steady state levels of deletion constructs were similar to those of full length Rnq1 (Figure S1A). Following transient co-expression of deletion constructs with full-length Rnq1 (to allow for transfer of the prion state), the maintainer plasmid was shuffled out, and the presence of prions composed of Rnq1 fragments, hereafter referred to as mini-[*PIN^+^*]s, was scored using the [*PSI^+^*] induction assay ([53],[69]; see [*PSI^+^*] Induction Assay in Materials and Methods).

The assay relies on the requirement of [*PIN^+^*] for the *de novo* formation of [*PSI^+^*] and requires that the strain carry a [*PSI^+^*]-inducing construct and a reporter for the detection of [*PSI^+^*]. Our strain carried the *SUP35NM::YFP* inducer, under the control of a tightly regulated *GAL1* promoter, and the *ade1-14* reporter, a premature stop in the chromosomal *ADE1* gene allowing to score the appearing [*PSI^+^*] colonies by their ability to grow on media lacking adenine. The Sup35NM::Yfp fusion was used as an auxiliary reporter during [*PSI^+^*] induction on galactose medium: its incorporation into newly forming [*PSI^+^*] aggregates allowed their visualization by fluorescent microscopy [70],[71].

Figure 1B demonstrates that, after elimination of full-length Rnq1, cultures expressing deletion constructs lacking any one QN region remained Pin^+^ (similar data for Δ2D and Δ3E not shown). The Pin^+^ phenotype was determined by mini-[*PIN^+^*]s, as the propagation of the phenotype required the presence of Rnq1 fragments: the loss of deletion plasmids was always accompanied by the loss of ability to become [*PSI^+^*] (12–18 Leu^−^ clones were analyzed for each construct; detailed analysis of mitotic stability of mini-[*PIN^+^*]s is presented later, in the section of Results including Figure 4). Also, Rnq1 fragments sustained the aggregated state: cells with bright fluorescent Rnq1::Cfp foci were detected in diploids from crosses of Pin^+^ cultures carrying Rnq1 deletion constructs with a [*pin^−^*] 64-D697 strain carrying pCUP-RNQ1::CFP (Figure 1C; hereafter this test is referred to as Rnq1::Cfp aggregation test).

The fact that fragments lacking QN1, QN2, QN3 or QN4 exist in an aggregated self-perpetuating state in the cultures not expressing wild type Rnq1 showed that no one QN region in Rnq1 is essential for prion maintenance. This raised an intriguing possibility of the redundancy in the prion-forming ability of QN-rich determinants. The alternative possibility, that the retained in all constructs Q-rich QG_10_ and/or a sequence upstream of QG_10_ are capable of maintaining [*PIN^+^*] even in the absence of QN regions, appeared highly unlikely based on earlier studies of N-terminal Rnq1 fragments [65]. It was further excluded by demonstrating that QG_10_ is not required for mini-[*PIN^+^*] establishment, and that the QN-rich C-terminus is indispensable for mini-[*PIN^+^*]s in our experimental setup (Figure S2). Thus, the [*PIN^+^*] prion domain is located in the QN-rich C-terminus, but no one QN region in Rnq1 is essential for the maintenance of the prion state, suggesting that Rnq1 prion domain carries multiple determinants capable of independently supporting the prion state.

### Multiple aggregation determinants in Rnq1

**Figure 2:**
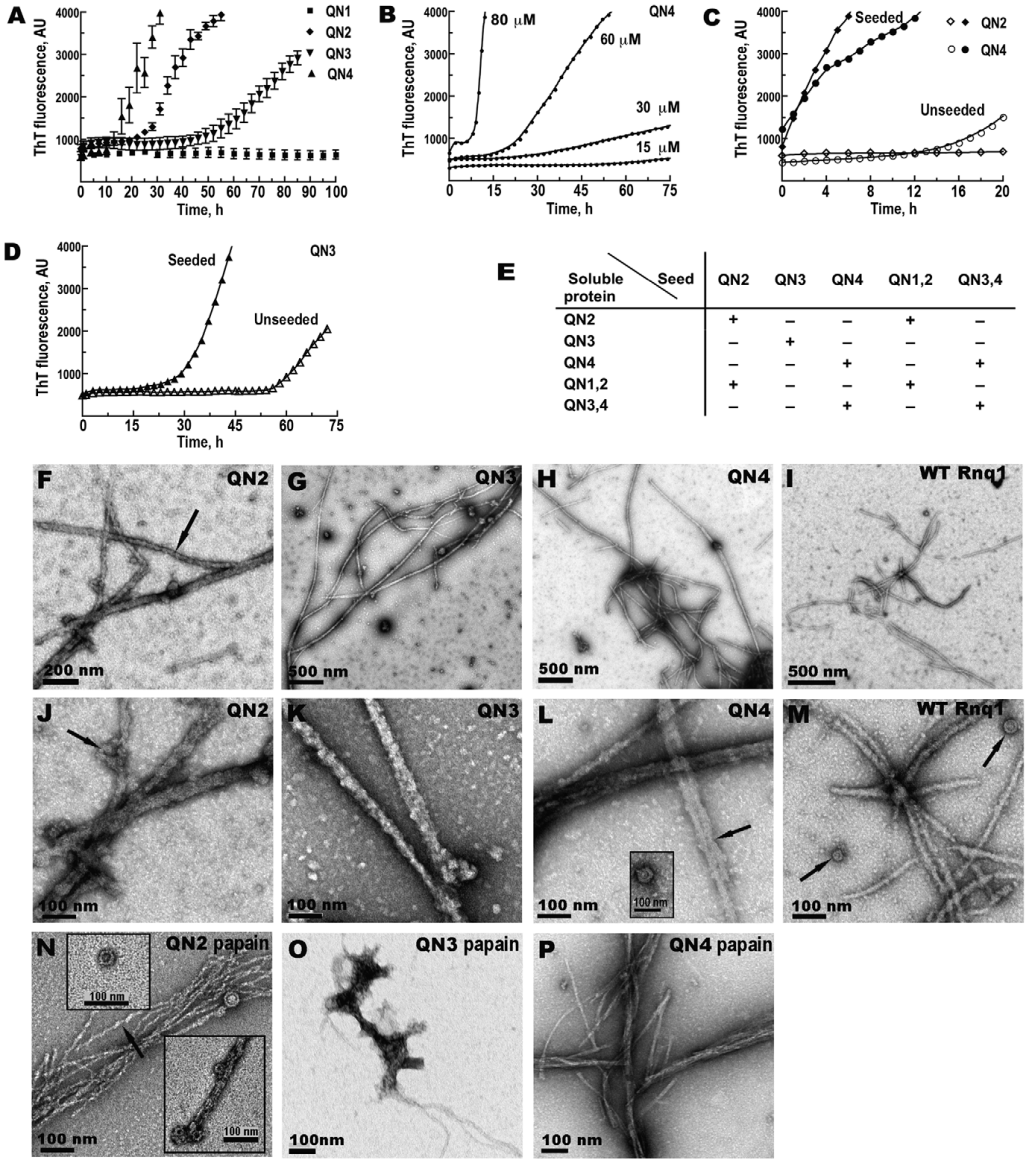
QN2 (ΔB1C2E4), QN3 (ΔB1C2E4), and QN4 (ΔB1C2D3), but not QN1 (ΔC2D3E4), can drive amyloid fiber formation *in vitro*. (A–D) Kinetics of *in vitro* aggregation of recombinant proteins monitored by ThT fluorescence. (A) Unseeded reactions. Concentrations: QN1 - 100 µM, QN2 - 70 µM, QN3 - 80 µM, QN4 - 70 µM. Shown are averages and standard deviations of 2-hour time points based on 3–5 independent experiments. (B) Concentration dependence of the lag phase for QN4 aggregation. (C) Elimination of lag phase in seeded reactions for QN2 and QN4. Concentrations of soluble proteins were 70 µM. (D) Retention of lag phase in seeded reactions for QN3. Concentration of soluble protein was 80 µM. (E) Summary of the analysis of cross-seeding between Rnq1 fragments carrying different QN regions (see Figure S3A). QN1,2 and QN3,4 correspond, respectively, to ΔD3E4 and ΔB1C2 in Figure 1A. (+) indicates disappearance or, in the case of QN3, reduction of the lag phase; (−) indicates no change in the kinetics of fiber formation upon the addition of seeds to soluble proteins. (F–P) Transmission electron micrographs of negatively stained fibers. In (N–P) fibers were treated with papain. Arrows show lateral association of fibers (L), ring-like oligomers (J, L inset, M, N insets) and twisted appearance of QN2 fibers (F,N).

To determine which QN regions could drive prion-like aggregation of Rnq1, bacterially expressed Rnq1 fragments lacking three out of four QN regions were tested for the propensity to form amyloid *in vitro*. Incubation of proteins encompassing only QN2 (ΔB1D3E4), QN3 (ΔB1C2E4) or QN4 (ΔB1C2D3) with Thioflavin T (ThT) resulted in a shift of the ThT excitation spectrum and increase of ThT fluorescence at 483 nm, indicative of amyloid formation (Figure 2A). Sigmoidal fluorescence kinetics was consistent with the presence of a rate-limiting nucleation step followed by a fiber growth phase. The QN1-bearing protein (ΔC2D3E4) did not form amyloid even at very high concentrations.

Aggregation kinetics of QN2 and QN4 was similar except that at equal protein concentrations the lag phase was slightly shorter for QN4. The threshold protein concentration for fiber formation was ∼15–20 µM, and the length of the lag phase was reproducible and concentration-dependent in the 30–80 µM range (Figure 2B and not shown). When reactions were seeded by preformed homologous fibers, the lag phase was completely eliminated (Figure 2C). In the cross-seeding reactions QN2 and QN4 could efficiently seed and be seeded by larger Rnq1 fragments, as long as they encompassed, respectively, QN2 and QN4 regions, implicating these regions in specific interactions during cross-seeding (Figure 2E and Figure S3A). For QN3, the threshold concentration was higher, ∼80µM, and the lag phase was considerably longer compared to QN2 and QN4 (Figure 2A), indicating that QN3 has a weaker aggregation propensity. The kinetics of seeded QN3 reactions was also distinct from QN2 and QN4: the QN3 fluorescence curve remained sigmoidal and a 10–20 hr lag phase was observed regardless of the amount of seed added (Figure 2D and data not shown). Such unusual kinetics was previously observed for the PrP_90-231_ fragment [72]. Also, QN3 was only capable of self-seeding, but could not template or be templated by a fragment including both QN3 and QN4 (QN3,4; Figure 2E and Figure S3A). QN3,4 aggregation could only be self-seeded or seeded by QN4 and there was no lag phase in these reactions (*ibid.*). This suggests that the conformation of the QN3 region in the fibers made from a Rnq1 fragment in which QN3 is the only aggregation determinant is different from conformations QN3 can take when combined with other QN regions.

Electron microscopy revealed networks of >1 µm-long fibers in QN2, QN3, and QN4 samples (Figure 2F–2H and 2J–2L). Fibers were ∼18–25 nm in diameter and unbranched, but frequently 2 or more fibers were associated laterally for part of their length producing thicker rope-like structures. In addition to fibers, we observed ring-like structures strikingly similar to oligomeric species previously seen during fiber formation by other amyloidogenic proteins [73],[74]; the oligomers were either lying separately or distributed irregularly along the fibers (Figure 2J and 2L inset). Similar fibers and oligomers were formed in the same conditions by full-length Rnq1 (Figure 2I and 2M).

As expected of amyloid, QN2 and QN4 fibers were protease-resistant. After hydrolysis with papain, they remained long, but became thinner (<10 nm in diameter). QN4 fibers became very smooth (Figure 2P), and QN2 fibers retained their twisted appearance (Figure 2N). Ring-like oligomers also remained and their structure became even more obvious (Figure 2N insets). Unexpectedly, papain treatment eliminated all long QN3 fibers, although occasional clusters of atypical aggregates with few protruding thread-like structures could still be detected (Figure 2O).

Thus, the Rnq1 prion domain encompasses three distinct QN-rich determinants that can independently drive aggregation of Rnq1 *in vitro*: QN2, QN3 and QN4. Among them, QN3 is weaker, and QN3 aggregates are somewhat atypical.

**Figure 3:**
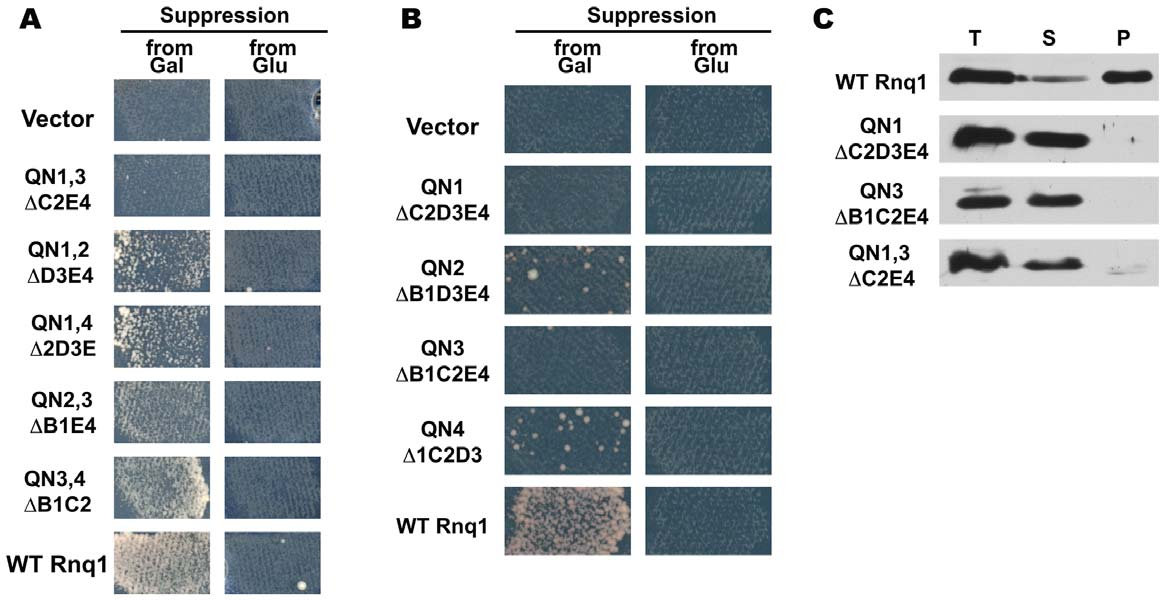
Transmission of the prion state to Rnq1 fragments with one or two QN regions. (A.B) The prion state can be transmitted from [*PIN^+^*] to Rnq1 fragments encompassing either QN2 or QN4. [*PSI^+^*] induction assay was performed as in Figure 1B. Shown is growth on SD-Ade after incubation at 20°C for 11 (A) and 13 (B) days. (C) Rnq1 fragments carrying only QN1 or/and QN3 do not co-aggregate with [*PIN^+^*]. Sedimentation analysis of the lysates of [*PIN^+^*] cells expressing both full-length Rnq1 and indicated deletion constructs; the WT Rnq1 and the QN1,QN3 panels show same lanes in the top and the bottom parts of the Western blot. See Figure S1B and S1C for steady state levels of Rnq1 fragments used in these experiments.

Lack of aggregation of QN1 suggests that its 12 aa long QN-rich stretch is not sufficient to drive aggregation of Rnq1 independently and confirms the lack of strong aggregation determinants upstream of QN1. However, using ThT fluorescence analysis and TEM, we demonstrated that the 12 aa peptide corresponding to QN1 region alone readily formed typical amyloid fibers (Figure 3SB and data not shown), suggesting that this region may function as aggregation determinant in the presence of other QN regions.

### The prion state can be transmitted to Rnq1 fragments carrying either QN2 or QN4

To test which QN regions could maintain prion state *in vivo*, *RNQ1* fragments encoding any one QN region or all possible combinations of two QN regions were substituted for the full-length *RNQ1* in the [*PIN^+^*][*psi^−^*] *rnq1-Δ* 74-D694 strain. As seen from the [*PSI^+^*] induction test, all fragments carrying two QN regions could be converted into mini-[*PIN^+^*]s as long as QN2 or QN4 were retained (Figure 3A; ΔB1D3 cultures were also Pin^+^ and in this test were similar to ΔB1C2 and ΔB1E4, not shown). Furthermore, low-level [*PSI^+^*] induction was detected even when using the constructs encompassing only QN2 or QN4 (Figure 3B). This weak Pin^+^ phenotype was confirmed using the Sup35NM::Yfp reporter: cells with bright foci indicative of [*PSI^+^*] appearance were readily detected in cultures expressing either QN2 or QN4, but not in the empty vector control (not shown).

On the contrary, cultures expressing Rnq1 fragments encompassing only QN1 and QN3, alone or together, became Pin^−^ after wild type *RNQ1* was shuffled out (Figure 3A and 3B; also no aggregate-containing cells were detected when Sup35NM::Yfp was used to screen for rare [*PSI^+^*]s). We also found no evidence that QN1 and QN3-bearing fragments were taking on a prion state that was incapable of inducing [*PSI^+^*]: these fragments remained soluble in [*PIN^+^*] cells before elimination of *RNQ1* (Figure 3C), and after the shuffle the lack of mini-[*PIN^+^*] aggregates was confirmed by a diffuse distribution of the reporter in the Rnq1::Cfp aggregation test (not shown).

Ability of Rnq1 fragments carrying either QN2 or QN4 to form mini-[*PIN^+^*]s is in agreement with *in vitro* data and further indicates that these regions represent independent prion determinants. Also, considering the specificity of cross-seeding of QN2- and QN4-encompassing fragments *in vitro* (Figure 2E), transmission of the prion state from [*PIN^+^*] to fragments carrying either QN2 or QN4 suggest that in the wild type [*PIN^+^*] prion both QN2 and QN4 regions are involved in prion formation and are available for templating. (To prove that the prion state was transmitted to Rnq1 fragments from the pre-existing [*PIN^+^*], and that Rnq1 fragments expressed from single-copy plasmids did not induce mini-[*PIN^+^*]s *de novo*, *RNQ1* deletion constructs were substituted for wild type *RNQ1* in [*pin^−^*][*psi^−^*] *rnq1-Δ* 74-D694, which resulted in Pin^−^ cultures; see Text S1). The more robust Pin^+^ phenotype observed when QN2 or QN4 were combined with either QN1 or QN3 compared to QN2 or QN4 alone (Figure 3A and 3B) suggests that QN1 and QN3 are also involved in prion formation. However, we found no indication that the prion state could be transmitted to fragments carrying only these QN regions. One possibility, consistent with the weak amyloid-forming propensity of QN1 and QN3 *in vitro*, is that *in vivo* their aggregation is contingent on the presence of QN2 or QN4.

### A barrier for the transmission of the prion state from [*PIN* ^+^] to mini-[*PIN* ^+^]s

As seen from Figure 3A and 3B, the Pin^+^ phenotype of cultures carrying shorter mini-[*PIN^+^*]s is notably reduced compared to the original [*PIN^+^*] strain. Furthermore, quantifying the frequency of [*PSI^+^*] induction using the Sup35NM::Yfp reporter revealed that deletion of any QN region except QN1 resulted in a drop in the induction of [*PSI^+^*] (Figure S4). Reduced [*PSI^+^*] induction could reflect (i) the appearance of [*pin^−^*] cells due to inefficient transmission of the prion state from the wild type [*PIN* ^+^], i.e. transmission barrier; (ii) accumulation of [*pin^−^*] cells because Rnq1 fragments were deficient in maintaining the prion state in the absence of full-length Rnq1; (iii) poor seeding of [*PSI^+^*] by mini-[*PIN* ^+^]s.

**Figure 4:**
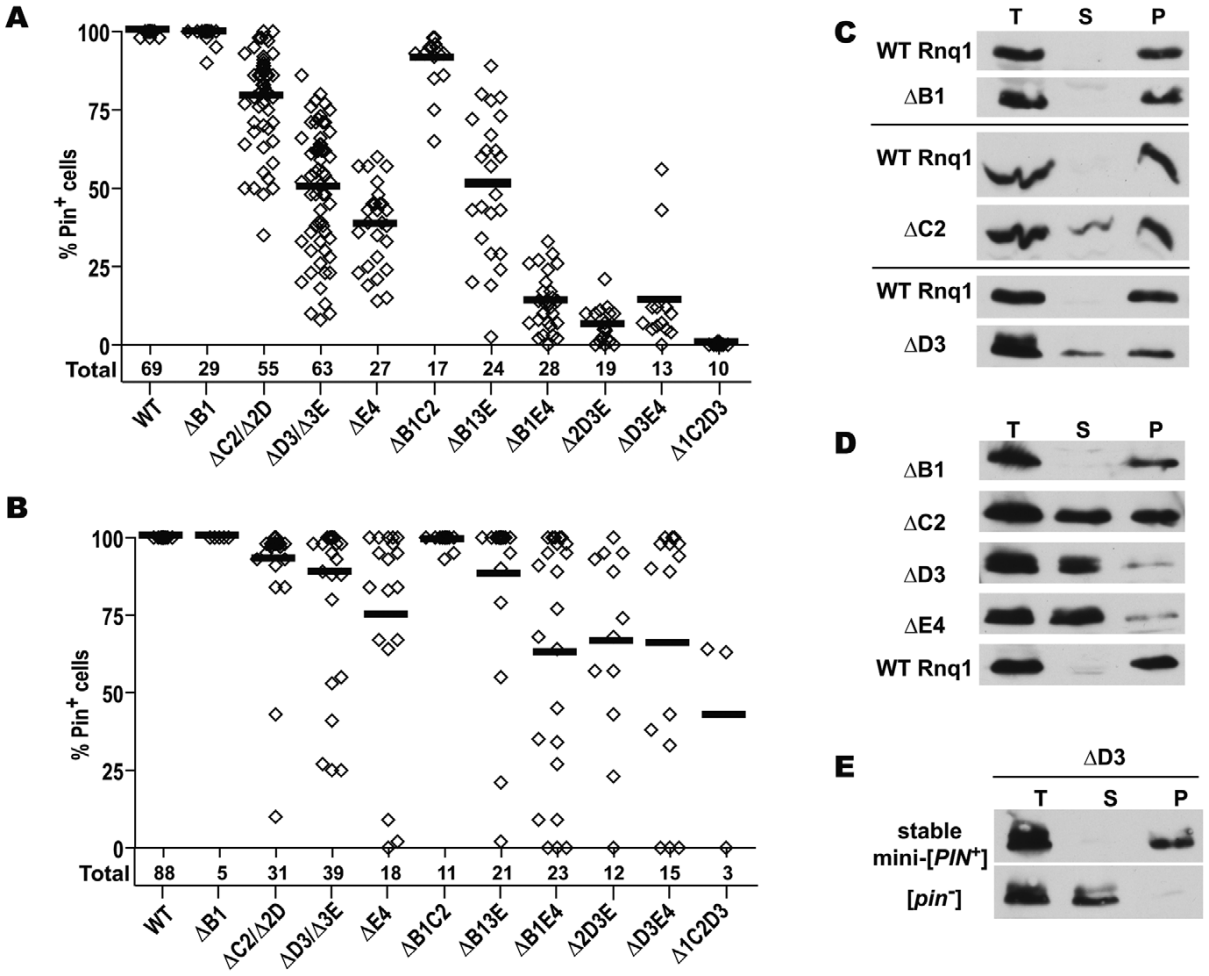
Transmission barrier for the conversion of Rnq1 fragments into mini-[*PIN^+^*]s by wild-type [*PIN^+^*]. (A) Substitution of *RNQ1* deletion constructs for full-length *RNQ1* in a [*PIN^+^*] strain leads to the appearance of [*pin^−^*] cells. Plasmid shuffle was performed as in Figure 1B. After selecting for the loss of full-length *RNQ1* on FOA, cultures now expressing only Rnq1 fragments were transferred to SD-Leu,His and then colony purified on this medium. 40 colonies from each culture were screened for the presence of mini-[*PIN^+^*]s *via* the [*PSI^+^*] induction test. Each data point represents the percentage of mini-[*PIN^+^*] colonies in one independent culture. The total number of cultures analyzed for each deletion is shown below the graph. Bars indicate average for all independent cultures. (B) Analysis of mitotic stability of mini-[*PIN^+^*]s reveals the presence of stable mini-[*PIN^+^*]s for all single and double deletions. Randomly chosen mini-[*PIN^+^*] isolates from the experiment described in 4A were passed once on SD-Leu,His and then colony purified again. Colonies from this second round of subcloning were analyzed as described in 4A. Several mini-[*PIN^+^*] isolates with high mitotic stability were also obtained for Δ1C2D3 in a separate experiment (MK and ID unpublished observations). (C–E) Sedimentation analyses of cell lysates. In [*PIN^+^*] cells still co-expressing wild-type Rnq1 (C) and after elimination of wild-type Rnq1 (D), ΔC2, ΔD3 and ΔE4 are detected in both soluble and aggregated fractions, whereas ΔB1 is aggregated. In (C) paired panels are from the same lanes at the top and the bottom of the Western blot. For (D), see Figure S7 for finer analysis of Δ2D and ΔE4 aggregation. (E) Rnq1 fragments are fully aggregated in stable mini-[*PIN^+^*] isolates. A ΔD3 mini-[*PIN^+^*] and a [*pin^−^*] colony were isolated from a ΔD3-expressing culture in experiment described in Figure 4A; stability of the mini-[*PIN^+^*] was determined as in Figure 4B.

To eliminate the last possibility as the only cause for weak Pin^+^ phenotypes in cultures expressing Rnq1 fragments, we analyzed the [*PIN*] status of individual cells in these cultures. After shuffling out the full-length *RNQ1*, the cultures were colony purified, and individual colonies were screened *via* the [*PSI^+^*] induction assay (see Figure S5 for a scheme of experiments). If reduced [*PSI^+^*] formation were exclusively due to poor seeding of [*PSI^+^*] by mini-[*PIN^+^*]s, a weak Pin^+^ phenotype was expected for all colonies. However, most cultures yielded both Pin^+^ and Pin^−^ colonies (Figure 4A) indicating that substitution of deletion constructs for full-length *RNQ1* lead to the loss of prion in some cells. Pin^−^ colonies were mitotically stable, and their [*pin^−^*] status was confirmed by the Rnq1::Cfp aggregation test (not shown) and, for several colonies, by sedimentation analysis (see e.g. Figure 4E). Although the proportion of [*pin^−^*] cells varied widely in cultures expressing the same Rnq1 fragment, there was a clear dependence of distributions upon the number and identity of QN regions (see Figure S6 for distributions). In most cases, the proportion of [*pin^−^*] cells correlated with the degree of reduction of [*PSI^+^*] induction prior to colony purification (compare Figure 4A and Figure S4). In agreement with genetic data, soluble Rnq1 was detected in the lysates of the cultures expressing all Rnq1 deletion constructs except ΔB1, and the proportion of soluble Rnq1 correlated with the percentage of [*pin^−^*] cells (Figure 4D, Figure S7, and data not shown).

We next asked if Rnq1 fragments were unable to form efficiently propagating mini-[*PIN^+^*]s, and if this deficiency in prion propagation could alone account for the accumulation of [*pin^−^*] cells, or there indeed was a barrier for the transmission of the prion state from [*PIN^+^*] to mini-[*PIN^+^*]s. The Pin^+^ colonies obtained by the colony purification described in the previous paragraph represent single-cell derived mini-[*PIN^+^*] isolates. To analyze mitotic stability of these mini-[*PIN^+^*]s, we passed them once on SD-Leu,His, then colony purified again and determined what proportion of colonies remained Pin^+^ (Figure 4B; see also Figure S5 for a scheme of experiments). Even though [*pin^−^*] cells did accumulate in a considerable proportion of mini-[*PIN^+^*] isolates, the data clearly indicate the existence of transmission barriers. Specifically, for all single and double QN deletions, there were mini-[*PIN^+^*]s that transmitted to all mitotic progeny (Figure 4B). Several such mini-[*PIN^+^*]s were selected for each construct and their high stability was confirmed by more extensive analysis (not shown). Furthermore, in these stable isolates, Rnq1 fragments were detected only in aggregated fractions (Figure 4E). This demonstrates that respective Rnq1 fragments are fully competent in maintaining the prion state. Also, for all deletion constructs, the average proportion of [*PIN^+^*] cells was much higher in mini-[*PIN^+^*] isolates than in original post-shuffle cultures, even though the number of cell divisions from a mini-[*PIN^+^*] cell to the second round of colony purification was no less than the number of divisions from the loss of full-length Rnq1 to the first round of colony purifications (compare Figure 4A and 4B and Figure S6). This indicates that the prion state was lost more frequently upon the elimination of full-length *RNQ1* or soon after. Our finding of stable and unstable mini-[*PIN^+^*] isolates may reflect the process prion strain formation, which is another hallmark of transmission barriers.

In a different approach we analyzed the aggregation of Rnq1 fragments in [*PIN^+^*] cells prior to the elimination of *RNQ1*. The rationale was that in the case of a transmission barrier Rnq1 fragments should remain partially soluble even in the presence of [*PIN^+^*]. Indeed, even ΔC2, ΔD3 and ΔE4 single deletions, for which fairly weak transmission barriers were indicated by genetic analysis, were detected in soluble fractions, and only ΔB1, for which the weakest barrier was seen, appeared fully aggregated (Figure 4C and not shown). For double and triple deletions, only ΔB1C2 was mostly aggregated, whereas other Rnq1 fragments remained mostly soluble in the presence of [*PIN^+^*], consistent with strength of respective transmission barriers (not shown).

Thus, we demonstrated that there is a barrier for the transmission of the prion state from [*PIN^+^*] to mini-[*PIN^+^*]s. None of the QN regions can ensure a barrier-free transmission, and elimination of any QN region results in the transmission barrier (a barrier towards ΔB1 seen in Figure 4A is very weak but was confirmed in other experiments; MK and ID unpublished observations).

### Contribution of oligopeptide repeats, hydrophobic patches, and QG_10_ to the transmission barrier

**Figure 5:**
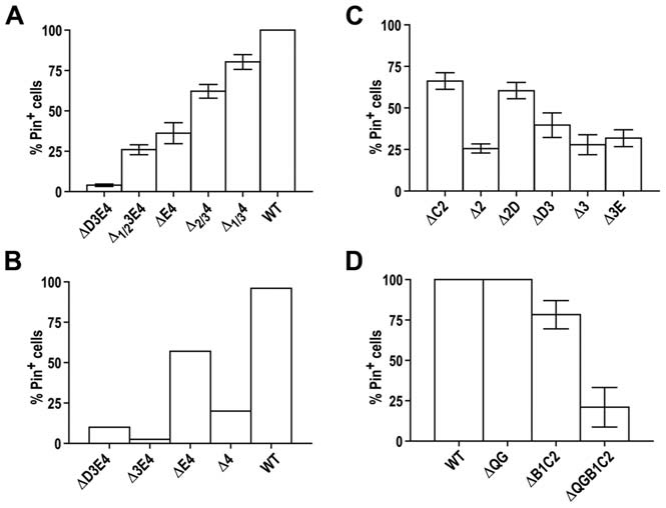
Contribution of parts of regions QN3 and QN4 (A), hydrophobic patches (B, C), and QG_10_ (D) to the loss of prion state upon substitution of Rnq1 fragments for full-length Rnq1. Experiments were performed as in Figure 4A. Bars show average for 6 (A), 2 (B), 3 (C), and 3 (D) independent experiments; standard error of the mean (SEM) is shown for (A,C,D). See Figure S1D for steady state levels of Rnq1 fragments and Figure S8 and Figure S9 for map, data and the analysis of mini-[*PIN^+^*]s formed by these fragments.

The complexity of Rnq1 prion domain goes beyond the presence of four QN-rich regions. Three QN regions, QN2, QN3 and QN4, encompass oligopeptide repeats (Figure 1A). To probe the contribution of these repeats to the transmission of the prion state, we tested if removing a part of a repeat-containing QN region is equivalent to its complete deletion. Experiments described in this section were performed with a set of C-terminal truncations gradually removing parts of regions QN3 and QN4 (Figure 5A and Figure S8). Comparison of Δ_1/2_ 3E4 (terminates right after the first oligopeptide of the QN3 repeat) with ΔD3E4, and of Δ_2/3_4 (the first oligopeptide of the QN4 repeat is preserved intact) with ΔE4, shows that retaining only the first oligopeptide of the repeat is enough to lower the transmission barrier compared to complete deletion of the respective QN region, but is not equivalent to retaining the whole QN region. This suggests that each oligopeptide contributes to prion formation, and that retaining the repeated structure is not essential for this contribution. The appearance of [*pin^−^*] cells even in the cultures expressing Δ_1/3_4 (retains the complete QN4 repeat but lacks the very C-terminal QN-rich stretch and the preceding non-QN-rich sequence) indicates that both the repeats and the unique part of QN4 contribute to the prion conformation. The gradual increase of the transmission barrier with progressing C-terminal truncations is consistent with observations of Vitrenko et al. [65] who noted an increase in the number of [*pin^−^*] cytoductants following the transmission of [*PIN^+^*] to a smaller set of Gfp-tagged C-terminally truncated Rnq1 fragments.

Another feature of Rnq1 prion domain is the alternating pattern of QN regions and hydrophobic patches (Figure 1A) with a QN-rich region at the C-terminus. Our analysis suggests the importance of the C-terminal location of the last QN region: compared to ΔE4 and ΔD3E4 ending with QN-rich regions, the proportion of Pin^+^ cells was sharply reduced in cultures expressing Δ4 and Δ3E4 fragments carrying hydrophobic patches at their C-termini (Figure 5B). The importance of alternating QN regions and hydrophobic patches is not as clear. For example, the proportion of mini-[*PIN^+^*] cells was lower in Δ2 cultures than in ΔC2 and Δ2D, but there was no significant difference between Δ3, ΔD3 and Δ3E (Figure 5C and Figure S9A and S9B).

Finally, QG_10_, which does not lead to a transmission barrier when deleted alone (Figure S2C and S2D), may contribute to barriers if deleted with other regions. Compared to ΔB1C2, more of ΔQGB1C2 remained soluble in [*PIN^+^*] cells, and after *RNQ1* was eliminated and wild type [*PIN^+^*] was lost, a higher proportion of cells became [*pin^−^*] (Figure 5D, Figure S9C and S9D and data not shown). In this aspect QG_10_ is similar to QN1, for which effects on transmission barriers are seen mainly in the context of larger deletions.

### Cooperative action of QN-determinants in prion conformation

As shown in the previous sections, prion domain of Rnq1 encompasses multiple QN-rich aggregation determinants. None of them is essential for the transmission of the prion state from [*PIN^+^*] to Rnq1 fragments, and analysis of transmission to Rnq1 fragments with different sets of QN regions indicates that in the wild type [*PIN^+^*] prion several QN regions are aggregated and can be used for templating. Yet, transmission from [*PIN^+^*] to essentially any Rnq1 fragment involves crossing a barrier, which is similar to the barriers observed during interspecies transmission of other prions. These barriers are hard to explain if [*PIN^+^*] just included several relatively autonomous aggregated regions. Indeed, single aa mismatches, always present in the case of interspecies transmissions, could interfere with the transmission at particular short regions, but there are no single amino acid mismatches between [*PIN^+^*] and Rnq1 fragments; rather, whole aggregation-prone domains are removed. We hypothesized that transmission barriers are determined by an overall prion conformation and that the [*PIN^+^*] prion conformation is a result of co-operative action of multiple determinants that can transmit the prion state independently of each other.

Our model for conformational organzation of [*PIN^+^*] and the mechanism of transmission barriers stipulates that transmission of the prion state between Rnq1 and its fragments involves templating by the exactly matching QN regions. Yet, having a common QN region capable of propagating the prion state is not sufficient for barrier-free transmission because all regions, including those not participating in templating, contribute to the overall conformation and, consequently, to conformational barriers. The conformation of the resulting mini-[*PIN^+^*] is also a product of co-operative action of all determinants present in the Rnq1 fragment and, consequently, is different from the original [*PIN^+^*], even though there is not a single amino acid mismatch in the templating region.

The following predictions can be made based on this model: (i) there should be a reciprocal barrier for the transmission of the prion state from mini-[*PIN^+^*]s to the full-length Rnq1; transmission of prion state between Rnq1 fragments (mini-[*PIN^+^*] to mini-[*PIN^+^*]) should also occur across barriers (ii) mini-[*PIN^+^*] to mini-[*PIN^+^*] transmission should require the presence of a common QN region; (iii) one common prion determinant should generally allow for some level of transmission; (iv) relative strength of mini-[*PIN^+^*] to mini-[*PIN^+^*] barriers may be different compared to the transmission from the original [*PIN^+^*], even if the same QN regions are involved in templating.

To test these predictions, we utilized stable mini-[*PIN^+^*] isolates obtained in experiments described in Figure 4B. *LEU2*-marked *RNQ1* fragment constructs were substituted for the *URA3*-marked ones. Then wild type *RNQ1* or various *RNQ1* deletion constructs were introduced by transformation, and plasmid shuffle experiment was performed as previously described (see Figure 1B).

**Figure 6:**
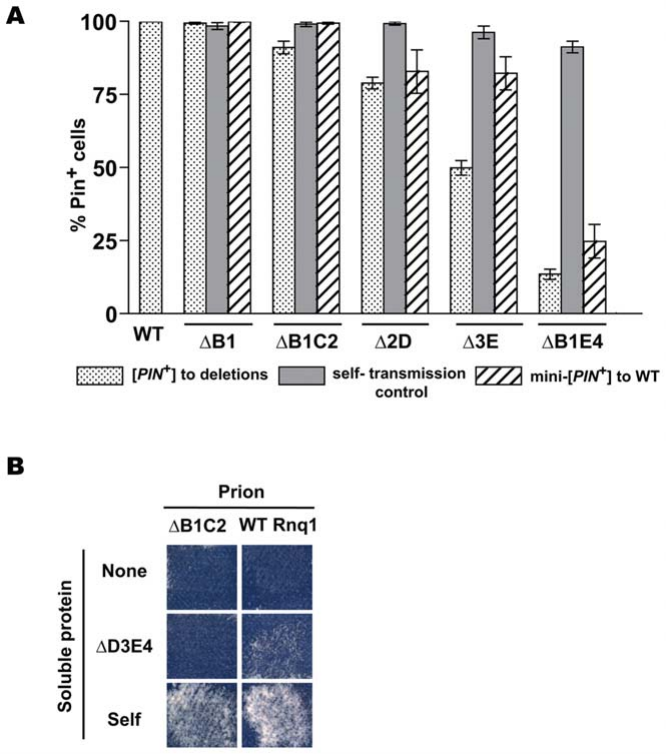
Cooperative action of QN regions determines transmission barriers for Rnq1-based prions. (A) Reverse transmission barriers for passing the prion state from mini-[*PIN^+^*]s to full-length Rnq1. Data for [*PIN^+^*] to mini-[*PIN^+^*] transmission are from Figure 4A. Reverse transmission from mini-[*PIN^+^*]s was analyzed the same way; shown are averages for 8–13 independent experiments and SEM. (B) Requirement of a common QN region for the transmission of prion state between Rnq1 fragments *in vivo*. Experiments were performed as in Figure 1B. Donors of prion state are indicated on top, recipients are listed on the left. Shown is growth on SD-Ade after 21 days at 20°C. Lack of transmission was confirmed by NM::Yfp and Rnq1::Cfp aggregation tests (not shown).

**Figure 7:**
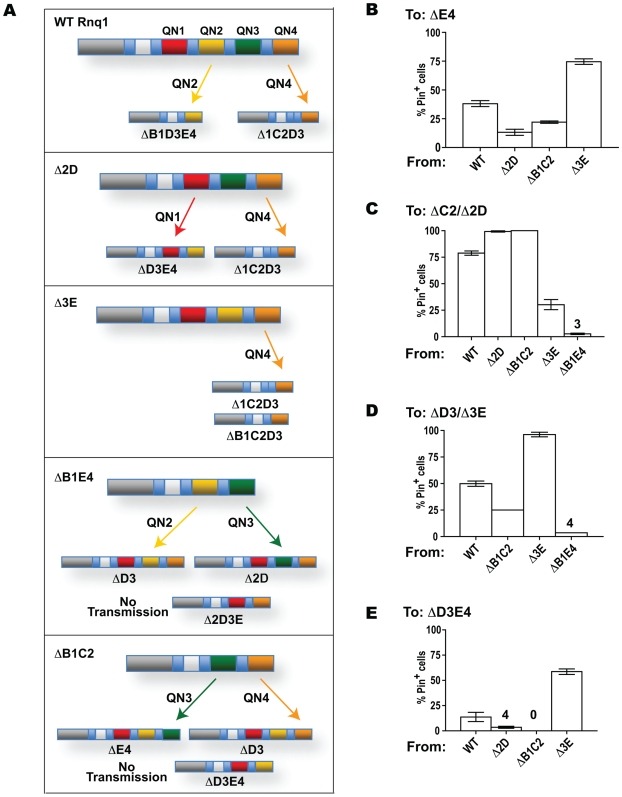
Transmission of the prion state between Rnq1 fragments. Experiments were performed as in Figures 1B and 4A. (A) Each of the QN regions can transmit the prion state. In the schematic diagrams of Rnq1 deletion fragments QN1, QN2, QN3 and QN4 are in red, yellow, green and orange, respectively. Arrows indicate possibility of transmission between the indicated constructs. The only common QN region responsible for templaring is indicated near each arrow. (B–E) Barrier strength for the transmission of the prion state to Rnq1 fragments depends upon what [*PIN^+^*] or mini-[*PIN^+^*] is templating the conversion. Data are grouped by the recipient Rnq1 fragments shown above the graphs, templating prions are listed under the graphs. Bars show averages of 3-12 independent experiments, SEM is shown for all datasets except the transmission from ΔB1C2 and ΔB1E4 to ΔD3, where only two experiments were performed. For strongest barriers averages are shown above the bars.

1. Figure 6A shows the existence of transmission barriers from mini-[*PIN^+^*]s towards full-length *RNQ1*, confirming that mini-[*PIN^+^*]s indeed represent prion conformational variants distinct from the original [*PIN^+^*] supported by full length Rnq1. The strength of the reciprocal barrier correlated with the strength of the transmission barrier from [*PIN^+^*] towards the respective Rnq1 fragment for most constructs (e.g. Δ2D and ΔB1E4), but there were several notable exceptions to this rule (e.g. Δ3E).
2. Importance of the presence of a common QN region was confirmed by transmitting the prion state from mini-[*PIN^+^*]s to other Rnq1 fragments. Figure 6B illustrates the lack of transmission between the ΔB1C2 mini-[*PIN^+^*] and the ΔD3E4 fragment. This result is in full agreement with *in vitro* data: ΔB1C2 (QN3,4) and ΔD3E4 (QN1,2) were unable to cross-seed each other while efficiently seeding QN4- and QN2-containing constructs, respectively (Figure 2E). Similarly, transmission was impossible from the ΔB1E4 mini-[*PIN^+^*] to Δ2D3E (not shown).
3. After establishing the specificity of QN-based templating, pairs of mini-[*PIN^+^*]s and Rnq1 fragments encompassing only one common QN region were used to demonstrate that each of the four QN regions could independently transmit the prion state. For QN2 and QN4, multiple examples of this ability were obtained, since the prion state could be transmitted to Rnq1 fragments encompassing only QN2 and QN4, respectively (Figure 7A; see also Figure 3B). Other examples include the transmission from ΔB1E4 mini-[*PIN^+^*] to ΔD3 for QN2-driven transmission, and from ΔB1C2 to ΔD3 for QN4-driven transmission (Figure 7A; data shown in Figure 7D). The ability of QN1 to template matching sequences in Rnq1-based prions was deduced from the transmission from Δ2D mini-[*PIN^+^*] to ΔD3E4 (Figure 7A; data shown in 7E), despite no transmission from ΔB1C2 to ΔD3E4 (see Figure 6B). Finally, templating by QN3 was shown by transmission from ΔB1E4 mini-[*PIN^+^*] to Δ2D (Figure 7A; data shown in 7C), despite no transmission from ΔB1E4 to Δ2D3E.
4. The strength of transmission barriers can be strikingly different when the same Rnq1 fragment is seeded by mini-[*PIN^+^*]s instead of [*PIN^+^*] (Figure 7B–7E). Changes in transmission barriers emphasize the leading role of QN2 and QN4 regions in Rnq1-based prions as removal of one of these regions increases the importance of the other. For example, compared to [*PIN^+^*], QN2-lacking mini-[*PIN^+^*]s Δ2D and ΔB1C2 are less efficient in converting ΔE4 (Figure 7B), and QN4-lacking ΔB1E4 mini-[*PIN^+^*] has a dramatically reduced transmission to ΔC2 (Figure 7C). The contribution of QN3 to the transmission barrier is also further underscored. When a Δ3E mini-[*PIN^+^*] is used for templating instead of [*PIN^+^*], the transmission barrier is simultaneously increased towards Δ2D (Figure 7C) and reduced towards the QN4-lacking ΔE4 and ΔD3E4 fragments (Figure 7B and 7E). It appears that QN3 modulates the contribution of QN2 and QN4 to the prion conformation and/or transmission, and elimination of QN3 increases the involvement of QN2 and decreases the involvement of QN4. The more complex contribution of the QN3 region to the conformation of [*PIN^+^*] is exemplified by the transmission to two similar constructs differing only in hydrophobic patch B: whereas [*PIN^+^*] or ΔB1C2 could template Δ1C2D3 but not ΔB1C2D3, Δ3E mini-[*PIN^+^*] transmitted prion state to both Δ1C2D3 and ΔB1C2D3 with similar efficiency (Figure 7A and data not shown).

## Discussion

### Multiple aggregation determinants of Rnq1

Together with [*PSI^+^*] and [*URE3*], [*PIN^+^*] belongs to the class of prions with QN-rich prion domains. The unique feature of Rnq1 is that it carries four QN regions separated by hydrophobic sequences (Figure 1A). Using a set of constructs retaining the entire N-terminal part of Rnq1 but bearing single and multiple deletions of QN regions in the C-terminus, we demonstrated *in vivo* and *in vitro* that prion domain of Rnq1 encompasses multiple prion determinants that can independently drive aggregation and transmit the prion state. Specifically, all four QN regions are able to transmit the prion state *in vivo*. Such transmission was demonstrated in experiments where the aggregated template and the recipient fragment had only one common QN region (Figure 7A). That transmission indeed occurred through the common QN sequences (that in our experimental system match exactly) was strongly indicated by two lines of evidence: (i) *in vivo* transmission and *in vitro* cross-seeding were completely blocked in the absence of common QN regions (Figure 2E, Figure 6B); (ii) the presence of one common QN region was generally sufficient for the transmission of the prion state to Rnq1 fragments that were otherwise known to maintain it.

Our data also suggest that, in Rnq1-based prions, multiple aggregation determinants simultaneously take on conformations allowing them to transmit the prion state. Indeed, in order to template a QN region in a soluble Rnq1 fragment, the corresponding QN region in a pre-existing [*PIN^+^*] (or mini-[*PIN^+^*]) has to be in a transmissible conformation, e.g. engage in a β-strand formation. And we found that [*PIN^+^*] and mini-[*PIN^+^*]s were each able to convert Rnq1 fragments carrying different sets of QN regions. For example, the ability of ΔB1E4 to convert ΔD3 and Δ2D indicates that in ΔB1E4 both QN2 and QN3 were in a transmissible state, and conversion by ΔB1C2 of ΔD3 and ΔE4 implies the transmissible state of both QN3 and QN4 in the ΔB1C2 mini-[*PIN^+^*] (Figure 7A). For the original [*PIN^+^*], transmission to constructs carrying only QN2 or only QN4 proves simultaneous prionization of these QN regions, whereas aggregation of QN1 and QN3 is suggested by different strength of transmission barriers to constructs lacking or retaining these regions (Figure 3 and Figure 7).

Even though all four QN regions can transmit the prion state, the contribution of these determinants to the maintenance of [*PIN^+^*] may not be equal. Indeed, *in vivo* mini-[*PIN^+^*] formation was confirmed for all fragments encompassing either QN2 or QN4, but was not detected for fragments carrying only QN1 and/or QN3, suggesting that QN1 and QN3 cannot maintain the prion state in the absence of QN2 or QN4. We strongly favor this explanation for QN1, as QN1 construct retaining the upstream part of Rnq1 was shown to have very low aggregation propensity *in vitro* (only a short QN1 peptide free of N-terminal part of Rnq1 was able to form fibers). Furthermore, QN1's inability to maintain the prion state is not unexpected, as the QN1 region corresponds to roughly a half of other QN regions in length, lacks oligopeptide repeats and has no Y residues that were hypothesized to facilitate the fragmentation of amyloid aggregates [75]. It is not so expected for the QN3 region, which is more similar to QN2 and QN4 in length and organization, and could independently drive Rnq1 aggregation *in vitro*, even though QN3 fibers formed after a notably longer lag phase and had atypical seeding kinetics and reduced protease resistance. So we also contemplated the possibility that the failure to obtain mini-[*PIN^+^*]s maintained by QN3 alone (or in combination with QN1) was due to extremely strong transmission barrier between [*PIN^+^*] and ΔB1C2E4 (or ΔC2E4, respectively). So far we were unable to bypass this presumptive barrier using mini-[*PIN^+^*]s as donors of the prion conformation (MK and ID, unpublished observations). For example, no transmission to ΔB1C2E4 or ΔC2E4 was detected from the ΔB1C2 mini-[*PIN^+^*], for which transmissible state of QN3 was indicated by the efficient conversion of ΔE4 (Figure 7A). Our finding that ΔB1C2E4 *in vitro* aggregation could not be seeded by fibers formed by QN3-containing fragments (Figure 2E) is consistent with either of the above explanations, as it could merely reflect the inability of the ΔB1C2E4 fragment to form “mature” amyloid or indicate a conformational barrier. The remaining possibility that QN1 and QN3-based mini-[*PIN^+^*]s are unable to facilitate the induction of [*PSI^+^*], thus making our most sensitive test for the transmission of the prion state non-informative, is a subject of our further explorations.

Our results provide a genetic framework for studies of the structure of [*PIN^+^*]. Out of several possible arrangements where multiple QN regions participate in β-strand formation, our data are most consistent with the one where all QN regions form parallel in-register β-sheets. This is in full agreement with a parallel in-register structure recently proposed for *in vitro*-made Rnq1 amyloid by Wickner et al. [66], who analyzed fibers formed by the Rnq1_153-405_ prion domain fragment by solid state NMR. Noteworthy, while Wickner et al. did not analyze the structure of different parts of the Rnq1 prion domain, their study utilized fibers with labeled Y residues, most of which are located in QN2, QN3 and QN4, indicating the applicability of the conclusion about parallel β-sheet structure to these QN regions. Another possible structure postulating aggregated state of several QNs is where QN regions of the same molecule interact with each other, e.g. pair-wise in a pseudo-dimer [Het-s]-like arrangement [4],[76]. Such structure appears unlikely for [*PIN^+^*]: contrary to the expectation of such arrangements, we found no evidence that aggregation of any QN region in Rnq1-based prions depended on the presence of another QN region. For example, we detected transmission of the prion state relying on the QN4 aggregation from Δ2D, Δ3E or ΔB1C2; transmission relying on QN3 from either ΔB1C2 or ΔB1E4; and transmission relying on QN2 from either Δ3E or ΔB1E4 (Figure 7A). Finally, structures where duplicated oligopeptides within repeat-carrying QN regions engage in intramolecular self-interactions [49] are also not indicated by our data, since deleting one of the two oligopeptides in a repeat was not equivalent to complete elimination of the repeated region (Figure 5A), and such equivalency is expected if repeated oligopeptides interacted.

Do other prions encompass multiple aggregation determinants that maintain a certain degree of independence? Such possibility is feasible for prions with complex prion domains, such as [*PSI^+^*], PrP^Sc^ and [Het-s]. Indeed, recent evidence indicates the presence of two distinct self-interacting regions within the Sup35 prion domain, one within the first 40 aa, and the other at ∼90–120 aa [34],[49],[50]. The second region appears to be expendable for the transmission of the prion state [48] and for fiber formation *in vitro* [77],[78], whereas expendability of the first region has never been tested directly. Similarly, four self-interacting regions have been identified for PrP [79]. The first of them, although not needed for prion replication, affects the structure of amyloid fibers [80], and the last, while being expendable for fiber formation [46], was hypothesized to be implicated in prion conversion [81]. For [*URE3*], the existence of a secondary prion-inducing region was considered upon the discovery of sequences in the non-prion domain part of Ure2, which facilitated/inhibited the *de novo* prion induction [82], but there is no evidence of multiple aggregation domains.

On the other hand, prion domain of Rnq1 stands out as the most complex among known prion domains. The redundancy of its structure is the result of several duplication events, with most recent duplications that created oligopeptide repeats in three out of four QN regions still traceable at the DNA level ([26] and our unpublished data). Considering that so far no cellular function has been assigned to non-aggregated Rnq1, and that [*PIN^+^*] was detected in industrial and pathogenic yeast isolates [24],[26], and has been shown to interact with prions and aggregation-prone proteins affecting formation, stability and toxicity of various amyloids (see Introduction), functional performance of the Rnq1 protein may involve formation of oligomeric complexes, if not [*PIN^+^*] itself. In this case redundant aggregation-prone sub-domains are likely to improve ability of Rnq1 to self-interact and broaden the repertoire of possible heterologous interactions.

### Transmission barriers in the absence of amino acid mismatches in transmitting regions

Transmission barriers were discovered upon attempting to transmit scrapie infectious agent to goats and mice [83]. The presence and strength of transmission barriers determines the possibility and efficiency of prion transmission between non-identical proteins, e.g. the risk of infecting humans with prions originating in wild and domestic animals. *In vitro* and *in vivo* studies of mammalian and yeast prions relate these barriers to species-specific differences in primary sequences of prion proteins [37],[46],[84],[85]. A search for the explanation of how differences in primary structure, and specifically single aa substitutions, can control the tightness of the transmission barriers led to the model based on the recognition element concept. According to this concept, transmission of the prion state requires the interaction between clearly defined critical regions, or recognition elements (see Introduction). Consequently, the model for transmission barriers postulates that non-matching aa residues within these regions may impose the barrier by disrupting the productive interaction of the recipient protein and the template [34],[79],[86],[87]. Thus, the strength of such barriers depends on the degree of sequence dissimilarity within this short region, and on the conformation of the recognition element in the template (i.e. the prion strain), which determines whether, and to what extent, a particular aa mismatch will affect the barrier. An absolute barrier is expected if the interaction is impossible, e.g. in the absence of a common recognition element. In our experimental system transmission is driven by four QN regions. While we do not explore the effects on transmission of individual aa mismatches within the common QN regions, the lack of transmission between Rnq1 fragments with no common QN regions could exemplify the absolute barrier mentioned above.

Yet, it would be an oversimplification to assume that all transmission barriers can be explained by primary structure dissimilarity in recognition elements [6],[88],[89]. Our study identifies and offers an explanation for transmission barriers not involving individual aa mismatches in recognition elements between the template and the recipient. We established that barriers exist for the transmission from full-length Rnq1 to Rnq1 fragments lacking any of the QN-rich aggregation determinants, and between all Rnq1 fragments encompassing non-identical sets of QN regions. At the same time, common QN regions, which drive the transmission across these non-absolute barriers, have fully matching sequences. Explaining these results in the framework of the abovementioned transmission barrier model is difficult, as it will involve postulating that deletion of any QN region changes the conformation of recognition elements in all other QN regions. However, our results are expected if overall conformation of [*PIN^+^*] and other Rnq1-based prions were a product of co-operative action of several aggregation domains. In this case a transmission barrier will form as a result of the inability of a recipient lacking some (or having extra) QN regions to take on exactly the same higher-order fold as the template. This distinct type of barrier will reflect a requirement for a conformational change at a higher level of amyloid structure despite conservation of identical primary structure and possibility of transmission within the recognition element(s) in common QN regions.

Upon further analysis of across-the-barrier transmission we gained additional support for the existence of transmission barriers determined by higher order conformational mismatches. (i) Since the recognition element is retained, such transmission barriers are likely to be non-absolute. Indeed, the presence of a common QN region was sufficient for the across-the-barrier transmission of the prion state to all constructs that were otherwise shown to be able to form mini-[*PIN^+^*]s. (ii) Also, while the interaction at the templating interface in the absence of aa mismatches may determine the same arrangement of the templating region in the newly forming prion, higher order conformation of this prion will be different. So prions forming by overcoming such transmission barriers are expected to have reverse barriers toward the proteins they were templated with. Indeed, reverse barriers were detected when transmitting the prion state from mini-[*PIN^+^*]s to full-length Rnq1; the strength of reverse barriers was not always reciprocal (Figure 5A). Importantly, inefficient reverse transmission is not predicted and was not observed for transmission barriers that are likely to be due to aa mismatches in recognition elements and where the transmission is presumed to occur through the selection of a recipient conformer compatible with the conformation of the template [6],[46],[47]. (iii) Recovery of both stable and unstable mini-[*PIN^+^*]s that indicates formation of prion strains during across-the-barrier transmission (MK and ID unpublished observations) is also expected when new prion folds are forming.

Some naturally occurring transmission barriers may be very similar to the ones described in our work, being based exclusively on higher-order conformations. For example, deletions and expansions of oligopeptide repeats are common both in yeast and human prion proteins. Rnq1 variants lacking oligopeptide repeats in QN3 and QN4 of Rnq1 were uncovered in a significant proportion of natural isolates [26]. Our data predict that these differences will impose barriers for the transmission of [*PIN^+^*] between yeast populations. The same study describes a Sup35 variant lacking two of the repeated oligopeptides. The Sup35 oligopeptide repeat region appears to be outside of recognition elements, but is involved in an ordered structure in the prion conformation [34], [49]–[51]. In this case previously demonstrated inefficient transmission of [*PSI^+^*] to Sup35 fragments with deletions in the oligopeptide repeat region can in part be due to transmission barriers. This possibility is consistent with a relatively high stability of “mini-[*PSI^+^*]” isolates originating from cultures, which were predominantly [*psi^−^*] after such transmissions [41]. Barriers determined by differences outside recognition elements may also play critical role in TSE epidemiology, specifically in limiting the spread of chronic wasting disease from cervides to other mammals. The 166–175 loop of PrP has been proposed to determine this transmission barrier, apparently without engaging in β-strand formation [90]. In summary, our work reveals the existence of transmission barriers in the absence of aa mismatches in transmitting regions and introduces the concept of distinct types of transmission barriers for complex prion domains. Forthcoming information on recognition elements for various prions should help determine the nature of established barriers and further elucidate their role in prion transmission.

## Materials and Methods

### Plasmids

Plasmids for expression of *RNQ1* and its deletion alleles in yeast were constructed on the backbone of pRS416 (*URA3*) or pRS415 (*LEU2*) *CEN* vectors; the *RNQ1* ORF and *RNQ1* fragments are controlled by the *RNQ1* promoter and followed by the *RNQ1* terminator sequence. To obtain bacterial expression constructs *RNQ1* fragments were amplified from corresponding yeast plasmids and cloned into pJC45 to yield N-terminally 10×HIS-tagged proteins. In *CEN HIS3*-marked pGAL-SUP35NM::YFP, the previously used Sup35NM::Yfp reporter [52] was placed under the control of the *GAL1* promoter. In *CEN URA3*-marked pCUP-RNQ::CFP, a fusion of complete wild type *RNQ1* ORF to *CFP* is controlled by the *CUP1* promoter. Plasmid construction and primers are described in Protocol S1 and Tables S1, S2, S3.

### Strains

Unless otherwise mentioned, all strains are derivatives of 74-D694 (*MAT* **a** *ade1-14 leu2-3,112 his3-Δ200 trp1-289 ura3-52*; [91]). The [*PIN^+^*][*psi^−^*] derivative is 1Y1 [48]. The [*pin^−^*][*psi^−^*] derivative is 1G4 [53], obtained from 1Y1 by GuHCl treatment [92]. The *rnq1*-Δ 74-D694 [*PIN^+^*][*psi^−^*] strain was constructed by seamlessly disrupting the complete *RNQ1* ORF using the integration-excision approach [93]. The pRS406-based *URA3* disrupting plasmid, pID130, contained the *RNQ1* promoter inserted as the *Eco*RI - *Bam*HI fragment (primers #19 and #3; see Table S3) and the sequence downstream of the *RNQ1* gene inserted as the *Sac*II - *Sac*I fragment (primers #31 and #32). During the disruption, the [*PIN^+^*] state was maintained by wild type *RNQ1* expressed from the *LEU2*-marked maintainer (see Plasmids). To facilitate subsequent plasmid shuffles, the *URA3*-marked maintainer was substituted for the *LEU2*-marked maintainer after disruption. To monitor the presence of [*PIN^+^*], the tightly regulated pGAL-SUP35NM::YFP *CEN HIS3* plasmid was introduced prior to the disruption and was maintained in the *rnq1*-Δ 74-D694 [*PIN^+^*][*psi^−^*] strain during subsequent experiments. The [*pin^−^*][*psi^−^*]version of *rnq1*-Δ 74-D694 was obtained from [*PIN^+^*][*psi^−^*]by transiently removing the *RNQ1* maintainer.

The [*pin^−^*][*psi^−^*] 64-D697 *MAT* **α** *ade1-14 leu2-3,112 lys9-A21 trp1-289 ura3-52* [53] was used in crosses to introduce pCUP-RNQ-CFP for the Rnq1::Cfp aggregation test.

### Yeast methods and cultivation procedures

Standard yeast media and cultivation procedures were used [94],[95]. Unless specifically mentioned, yeast were grown at 30°C on solid synthetic glucose media (SD) selective for plasmid maintenance. Cultures for transformation and protein isolation were grown in liquid organic complete YPD medium at 30°C with constant orbital agitation at 200 rpm. The *GAL* promoter was induced on synthetic media with 2% galactose as a single carbon source (SGal). The *CUP* promoter was induced on synthetic media supplemented with 20µM CuSO_4_. Media supplemented with 5-fluoroorotic acid was used for selective elimination of *URA3*-marked plasmids [96].

### [*PSI* ^+^] induction assay

The [*PSI^+^*] induction assay relies on the requirement of [*PIN^+^*] for the *de novo* formation of [*PSI^+^*] and requires that the strain carry a [*PSI^+^*]-inducing construct and a reporter for the detection of [*PSI^+^*] ([53]; reviewed in [69]). [*PSI^+^*]-inducing constructs expressing the prion domain of the [*PSI^+^*]-forming protein, Sup35, allow for the increase of [*PIN^+^*]–dependent appearance of [*PSI^+^*] to readily detectable levels. In our experiments, the strain carried the *SUP35NM::YFP* fusion under the control of a tightly regulated *GAL1* promoter. The promoter remained repressed while yeast were growing on glucose media prior to shuffling out the wild type *RNQ1* maintainer. After elimination of full-length *RNQ1*, expression of pGAL-SUP35NM::YFP was turned on by transferring yeast to galactose medium. To allow for the detection of [*PSI^+^*], the strain carried the *ade1-14* reporter, a premature stop codon in the chromosomal *ADE1* gene. The *ade1-14* mutation made the original [*PIN^+^*][*psi^−^*] *rnq1-Δ* 74-D694 strain unable to grow on media lacking adenine. However, in [*PSI^+^*] cells that appeared following *SUP35NM::YFP* overexpression, translation termination was compromised due to Sup35 aggregation, which resulted in nonsense suppression, i.e. occasional readthrough of the stop codon detectable as slow growth on -Ade.

Indicated *LEU2*-marked constructs were transformed into [*psi^−^*][*PIN^+^*] *rnq1-Δ* 74-D694 carrying a *URA3*-marked *RNQ1* maintainer and a *HIS*-marked pGAL-SUP35NM::YFP [*PSI^+^*]-inducer. Transformants were selected on SD-Leu,Ura,His and then passed twice on SD-Leu,His to allow for the loss of the maintainer. Ura^−^ cells were selected on FOA and transferred to SGal-Leu,His to induce [*PSI^+^*]. From galactose medium, yeast were replica plated to [*PSI^+^*] scoring media, SD-Ade and SEt-Ade (synthetic media containing, respectively, 2% glucose or 2% ethanol as a single carbon source) and to SD-Leu,His (growth control). SD-Ade and SEt-Ade plates were incubated at 20°C and 30°C and scored several times between days 5 and 25. In the control experiment yeast were grown on non-inducing SD-Leu,His prior to suppression analysis. Ade^+^ colonies were further confirmed to be [*PSI^+^*] by the GuHCl test [92], which was performed exactly as described in Derkatch *et al* [18] on media supplemented with 5 mM guanidine hydrochloride (GuHCl).

Additionally, [*PSI^+^*] induction was monitored by fluorescent microscopy of cultures grown on galactose medium using the Sup35NM::Yfp [*PSI^+^*]-inducer as a reporter: incorporation of Sup35NM::Yfp into newly forming [*PSI^+^*] aggregates allowed their visualization by fluorescent microscopy as characteristic ring-shaped structures [70],[71].

### Rnq1::Cfp aggregation assay

The assay (reviewed in [69]) relies on the ability of fusions of prion proteins with fluorescent reporters to join prion aggregates and allow their visualization [70]. The Rnq1::Cfp reporter was introduced by crossing *rnq1*-Δ 74-D694 cultures carrying *LEU2*-marked *RNQ1* deletion constructs with the [*pin^−^*] 64-D697 strain carrying the *URA3*-marked pCUP-RNQ1::CFP plasmid. Diploids were selected on SD-Ura,Leu. The reporter was induced by supplementing SD-Ura,Leu with 20µM CuSO_4_. Presence of cells with bright fluorescent foci was indicative of mini-[*PIN^+^*]s.

Note: moderate short-term (2–3 days) overexpression of Rnq1::Cfp does not induce the *de novo* appearance of [*PIN^+^*] in [*psi^−^*] strains, which were used in our experiments [19]. To confirm lack of *de novo* induction of [*PIN^+^*] in the diploids, the *rnq1*-Δ 74-D694 carrying empty vector instead of *RNQ1* deletion constructs was included in all crosses.

### Rnq1 sedimentation assays

Yeast cell lysates were prepared as described in [69] except that pre-clearing at 10,000×g was omitted. 60 µg of total protein were centrifuged at 280,000×g for 30 min at 4°C (Beckman Optima TLX centrifuge, TLA 120.2 rotor). After removing the supernatant (S), the pellet fraction (P) was resuspended in the protein extraction buffer. The S and P fractions and 60 µg of total lysate (T) were separated by SDS-PAGE. Rnq1 was detected by a Western blot with polyclonal antibodies raised against full-length Rnq1 (Type 2, a generous gift from S. Lindquist, Whitehead Institute; Figure S2D) or the N-terminal Rnq1 fragment (Rnq1A, kindly provided by E. Craig, University of Wisconsin-Madison; [97]; Figure 3C, Figure 4C–4E, Figure S1, and Figure S2B).

### Recombinant protein purification

*Escherichia coli* BL21-AI One Shot cells (Invitrogen) transformed with pJC45-based expression constructs (see Plasmids and Table S2) were cultured in LB medium supplemented with 100 µg/ml ampicillin at 37°C. Protein expression was induced at mid-log phase (OD_600_∼0.4) by 1mM IPTG and 0.2% L-arabinose for 1.5 h. Cells were harvested by centrifugation (4°C, 1600×g) and either processed immediately or frozen at −80°C. Due to limited solubility of Rnq1 in aqueous solutions, purification was carried out under denaturing conditions at room temperature. Cells were lysed by gentle agitation in lysis buffer (100mM NaH_2_PO_4_ pH 7.4, 350mM NaCl, 8M urea) for 1h. Cell debris was removed by centrifugation at 20,000×g for 10 min. Supernatant was incubated for 2 h with Ni-NTA Sepharose (Qiagen) equilibrated with lysis buffer. The slurry was transferred to the column and washed extensively with lysis buffer. Rnq1 was eluted with lysis buffer containing 250mM imidazole. Protein enriched fractions were determined by UV absorption at 280nm and concentrated on Centricons (Millipore). Protein concentration was determined by the BCA assay (Pierce). Purity of recombinant proteins was estimated by SDS-PAGE as >95%.

### *In vitro* fiber formation

The 200 µl reactions were set up in assembly buffer (1M urea, final concentration; 100mM NaH_2_PO_4_ pH 7.4; 300mM NaCl) in the presence 5 µM ThT. Fiber formation was monitored by ThT fluorescence [98] in a Molecular Devices SpectraMax M-5 plate reader (λ*_ex_* 450 nm; λ*_em_* 483 nm; 24°C). Readings were taken every 15 min, samples were shaken for 5 sec prior to each reading. For each protein, experiments were performed in duplicate and repeated at least 3 times. To obtain fibers for seeded reactions, suspensions of polymerized Rnq1 fragments were collected from the wells and precipitated with 5 volumes of Met-OH. Pellets were washed 3 times with 70% Et-OH to remove urea, and then dried with anhydrous acetone. Seed powder was stored at 4°C and dissolved in the assembly buffer right before adding to the samples. Papain digestions were performed at 25°C for 30 min. Papain (Sigma) was added directly to ThT reactions (enzyme:substrate ratio 1∶50).

### Fluorescence microscopy

Cells were observed using an Axioplan2 Zeiss microscope. Images of representative fields were captured with a Zeiss Axiocam digital camera and processed with Improvision OpenLab software. Fluorescence and differential interference contrast images (DIC) are shown for each field.

### Transmission electron microscopy (TEM)

TEM was performed at the NYU School of Medicine Image Core Facility. Fiber suspensions in the assembly buffer were diluted 2.5-fold in water and 4 µl were applied onto the carbon-coated 400 mesh Cu/Rh grids (Ted Pella Inc.). The grid was washed 3 times with water to get rid of urea, and negatively stained with 1% uranyl acetate (twice briefly and then for 5 min at 25°C). Images were obtained using Philips CM12 transmission electron microscope supplied with a Gatan 1k×1k digital camera and processed using Gatan Digital Micrograph software.

## Supporting Information

Figure S1 Expression of Rnq1 fragments in yeast. Western blot analysis of the lysates of [*PIN^+^*][*psi^−^*] *rnq1-Δ* 74-D694 cells co-expressing Rnq1 and the indicated deletion constructs. Both full-length *RNQ1* and its fragments are controlled by the native *RNQ1* promoter (see Plasmids in Materials and Methods, and Protocol S1 for plasmid construction). Transformants were maintained on the medium selective for both plasmids, but cultures for protein isolation were grown in YPD. Yeast cell lysates were prepared as described in Liebman et al. [2006] except that pre-clearing at 10,000×g was omitted. Rnq1 was detected with polyclonal antibodies raised against the N-terminal part of the protein ([Lopez et al., 2003]; kindly provided by E. Craig, University of Wisconsin-Madison). Panels show same lanes in the top and bottom parts of the Western blot of the same culture. All Rnq1 fragments ran in accordance with their expected size. At least 2 independent transformants were analyzed for each construct and experiments were repeated 2–5 times. The groups are: deletions of one (A), two (B) and three (C) QN regions with preceding hydrophobic patches used throughout the manuscript; and (D) other constructs used mostly in Figure 5, Figure S2, Figure S8, and Figure S9. (Liebman SW, Bagriantsev SN, Derkatch IL (2006) Biochemical and genetic methods for characterization of [*PIN^+^*] prions in yeast. Methods 39: 23–34.) (Lopez N, Aron R, Craig EA (2003) The role of Sis1 on the maintenance of [*RNQ^+^*] prion. Mol Biol Cell 14: 1172–1181.)
(3.22 MB TIF)

Figure S2 The QN-rich C-terminus is an essential part of the prion domain of Rnq1. N-terminal Rnq1 fragments were previously shown to be unable to join [*PIN^+^*] or to transmit the prion state [Vitrenko et al., 2007]. However, in those studies Rnq1 fragments were Gfp-tagged, and large tags sometimes interfere with prion properties [Dagkesamanskaia et al., 1997; Edskes et al., 1999]. We confirm that the QN-rich C-terminus is indispensable for mini-[*PIN^+^*]s in our experimental setup, and demonstrate that QG_10_ is not required for mini-[*PIN^+^*] establishment. (A,B) ΔB1C2D3E4, a Rnq1 fragment lacking all QN regions but retaining QG_10_, does not aggregate in [*PIN^+^*] cells and does not carry on the Pin^+^ phenotype. (A) Cultures expressing ΔB1C2D3E4 become Pin^−^ upon the loss of full-length Rnq1. Plasmid shuffle and [*PSI^+^*] induction test were performed as described in Figure 1B legend. The lack of [*PSI^+^*] formation was confirmed by fluorescent microscopy using the Sup35NM::Yfp reporter (not shown). (B) There is no evidence of ΔB1C2D3E4 aggregation even in the presence of [*PIN^+^*]. Sedimentation analysis of the lysate of [*PIN^+^*] cells co-expressing Rnq1 and ΔB1C2D3E4; panels show same lanes in the top and bottom parts of the Western blot of the same culture. Similar data for Δ1C2D3E4 not shown. (C,D) QG_10_ is not essential for maintaining the prion state of Rnq1: ΔQG is aggregated, and cultures remain Pin^+^ after elimination of Rnq1. (C) Plasmid shuffle and [*PSI^+^*] induction test were performed as described in Figure 1B legend. (D) Sedimentation analysis of cell lysates from cultures expressing indicated fragments after elimination of full-length Rnq1. Similar data for ΔAQG not shown. (Vitrenko YA, Pavon ME, Stone SI, Liebman SW (2007) Propagation of the [*PIN^+^*] prion by fragments of Rnq1 fused to GFP. Curr Genet 51: 309–319.) (Dagkesamanskaia AR, Kushnirov VV, Paushkin SV, Ter-Avanesyan MD (1997) Fusion of glutathione S-transferase with the N-terminus of yeast Sup35 protein inhibits its prion-like properties. Genetika (Rus) 33: 610–615.) (Edskes HK, Gray VT, Wickner RB (1999) The [*URE3*] prion is an aggregated form of Ure2p that can be cured by overexpression of Ure2p fragments. Proc Natl Acad Sci USA 96: 1498–1503.)
(3.74 MB TIF)

Figure S3 *In vitro* analysis of aggregation of Rnq1 protein fragments and QN1 peptide. (A) Cross-seeding between Rnq1 protein fragments lacking two or three QN regions. Kinetics of *in vitro* aggregation was monitored by ThT fluorescence. The soluble protein is indicated above the graphs, and seeds are listed to the right in brackets. “No seed” indicates unseeded polymerization. Proteins used: QN2 (ΔB1D3E4), QN3 (ΔB1C2E4), QN4 (ΔB1C2D3), QN1,2 (ΔD3E4), QN3,4 (ΔB1C2). Concentrations of soluble proteins were in the 60–80 µM range. (B) Transmission electron micrographs of negatively stained QN1 peptide fibers. The 12 aa long QN1 peptide (NSNNNNQQGQNQ; GenScript; 98.8% purity) was pre-treated with 1,1,1,3,3,3,-hexafluoro-2-isopropanol (Sigma) for 24 h at room temperature and lyophilized. The powder was re-suspended in water to a final concentration of 250 µg/ml. The 200 µl reactions were set up in the presence of 5 µM ThT. Samples incubated at 37°C for ∼80 h were shaken for 5 sec every 10 min. Monitoring the aggregation kinetics by ThT fluorescence (see Materials and Methods) revealed a sigmoidal curve with a very short lag phase (not shown). TEM was performed at the NYU School of Medicine Image Core Facility as described in Materials and Methods. Long and very thin (<10 nm in diameter) fibers were frequently laterally associated and had either straight or twisted appearance.
(5.34 MB TIF)

Figure S4 Reduction of the *de novo* formation of [*PSI* ^+^] after substitution of deletion constructs for full-length *RNQ1*. Plasmid shuffle was performed in the [*PIN* ^+^][*psi* ^−^] *rnq1*-Δ 74-D694 strain carrying the pGAL-SUP35NM::YFP [*PSI* ^+^] inducer as described in Figure 1B legend. [*PSI* ^+^] was induced by growth on SGal-Leu,His plates for 3 days. Relative levels of [*PSI* ^+^] induction were calculated by determining the percentage of cells with Sup35NM-Yfp fluorescent aggregates in cultures expressing indicated constructs, and then normalizing it to the percentage of aggregate-containing cells in cultures carrying wild-type [*PIN* ^+^] only. Each data point represents an independent experiment, in which the percentage of [*PSI* ^+^] cells determined for three transformants expressing the deletion construct is normalized to the percentage of [*PSI* ^+^] cells in three transformants expressing wild-type Rnq1 (a total of 500–1,000 cells were analyzed in each case; wild-type [*PIN* ^+^] cultures carried 30–40% aggregate containing cells). Data for constructs ΔC2 and Δ2D, and ΔD3 and Δ3E were similar and are grouped. Among the constructs lacking only one of the four QN regions, elimination of QN4 had the biggest effect reducing [*PSI^+^*] induction ∼5-fold, whereas deleting QN2 or QN3 reduced it ∼2-fold. The effect of double deletions varied depending upon what QN regions were eliminated. Eliminating QN3 in conjunction with a lack of either QN2 or QN4 led to an almost 100-fold drop in [*PSI^+^*] induction, whereas deletion of QN1 only mildly increased the effect of eliminating QN3 and QN4 and, surprisingly, had a rescuing effect when deleted together with QN2. Finally, the level of [*PSI^+^*] induction was the lowest in cultures expressing the Rnq1 fragments with only one QN region.
(2.38 MB TIF)

Figure S5 Scheme of the analysis of the transmission of the prion state from [*PIN* ^+^] to Rnq1 fragments. Experiments are described in the text, Materials and Methods and Legends for Figure 1B and 1C, Figure 4A and 4B. Experiments described in Figure 6 and Figure 7 were performed similarly except the initial strain expressed a Rnq1 fragment and harbored a mini-[*PIN* ^+^], and the prion state was transmitted to wild-type Rnq1 or to other Rnq1 fragments.
(0.57 MB TIF)

Figure S6 Distributions of percentages of mini-[*PIN* ^+^] cells in cultures expressing indicated Rnq1 fragments after the loss of wild-type [*PIN* ^+^]. Analysis of data presented in Figure 4A (Round 1 colony purification) and in Figure 4B (Round 2 colony purification). Horizontal axes show percentage of [*PIN* ^+^] cells in 10% increments. Vertical axes show frequency of cultures with corresponding percentages of mini-[*PIN* ^+^] cells.
(8.31 MB TIF)

Figure S7 After elimination of full-length Rnq1, Δ2D, and ΔE4 were detected in both soluble and aggregated fractions. For finer analysis of aggregates formed by Rnq1 fragments, 0.5 ml (∼1 mg) of total protein were loaded onto ∼4.5 ml of 15%–40%–60% step sucrose gradient and centrifuged at 160,000×g for 60 min at 4°C (Beckman Optima L-90K centrifuge, SW55Ti rotor). The 0.5 ml fractions were collected from the bottom of the tube, resolved on SDS-PAGE and immunoblotted with anti-Rnq1A. The bottom fraction is not shown. Predominately aggregated wild-type Rnq1 is not detected in the top (soluble) fraction, whereas partially soluble Δ2D and ΔE4 are detected in the top fraction, as well as in the same gradient fractions were WT Rnq1 is present. Consistent with Figure 4A and 4D, ΔE4 lysates have more soluble Rnq1 fragment than Δ2D lysates.
(2.17 MB TIF)

Figure S8 Transmission barrier for conversion of Rnq1 fragments lacking parts of regions QN3 and QN4 into mini-[*PIN* ^+^]s. (A) Schematic diagram of Rnq1 and deletion constructs used here and in Figure 5A. QN-rich regions are in red; patterned blocks within QN regions indicate oligopeptide repeats; hydrophobic patches are in blue. Lines indicate regions present, and nomenclature refers to deleted regions. Δ_1/3_4 retains the oligopeptide repeat of QN4 but lacks the very C-terminus of Rnq1 that includes a non-QN-rich and a QN-rich stretch. In Δ_2/3_4 only the first oligopeptide of the QN4 region is preserved intact. Δ_1/2_3E4 terminates right after the first oligopeptide of the QN3 repeat. (B) Percentage of mini-[*PIN* ^+^] cells in cultures bearing indicated deletion constructs after wildtype [*PIN* ^+^] loss. See Figure 4A legend for full description of the experiment. (C) Analysis of mitotic stability of mini-[*PIN* ^+^]s formed by the indicated fragments. Data points show percentage of mini-[*PIN* ^+^] cells in clonal mini-[*PIN* ^+^] isolates after ∼20 generations of mitotic growth. See Figure 4B legend for full description of the experiement. The total number of independent cutures (B) or mini-[*PIN* ^+^]s (C) analyzed for each deletion construct is indicated on each graph. Bars indicate averages. In (B) note the gradual decrease in the proportion of mini-[*PIN* ^+^] cells in cultures with progressive truncations of each QN region. Significant differences between WT Rnq1 and Δ_1/3_4; Δ_1/3_4 and Δ_2/3_4; and Δ_2/3_4 and ΔE4 show that the C-terminal part of QN4, as well as each of the repeated peptides contribute to the transmission barrier (see SEM in Figure 4F). The significant difference between Δ_1/2_3E4 and ΔD3E4 illustrates the contribution of the first of the repeated peptides in QN3. Data in (C) shows that stable mini-[*PIN* ^+^]s can be obtained after transmission from [*PIN* ^+^] to any of the fragments.
(6.99 MB TIF)

Figure S9 Analysis of the importance of non-QN-rich sequences within the C-terminal part of Rnq1. (A,B) Analysis of the importance of alternating QN regions with hydrophobic patches in the prion domain in Rnq1. (C,D) The QG_10_ deletion enhances the transmission barrier for the conversion of the Rnq1 fragment lacking QN1 and QN2. (A,C) Percentage of mini-[*PIN* ^+^] cells in cultures bearing indicated deletion constructs after wildtype [*PIN* ^+^] loss. (B,D) Analysis of mitotic stability of mini-[*PIN* ^+^]s formed by the indicated Rnq1 fragments. See Figure 4A and 4B and Figure S8 legends for full description of the experiments. In (A,B) the total number of independent cutures or mini-[*PIN* ^+^]s analyzed for each deletion construct is indicated on each graph. In (C,D) 3–5 independent cutures or mini-[*PIN* ^+^]s were analyzed for each deletion construct. In (A), note the significant reduction of prion-containing cells in the cultures expressing the Δ2 Rnq1 fragment (alteration of QN regions and hydrophobic patches is disrupted and hydophobic regions C and D are located next to each other), compared to ΔC2 and Δ2D (alternating pattern is preserved). Analysis of mitotic stability of mini-[*PIN* ^+^]s formed by these fragments in (B) indicates that the alternating pattern is important for the establishment of stable mini-[*PIN* ^+^] strains. Yet, although not part of the experiment shown in (B), stable mini-[*PIN* ^+^] isolates were obtained for Δ2, confirming the ability of this fragment to faithfully maintain the prion state (MK and ID unpublished). For Δ3, the reduction in prion containing cells in post-transmission cultures (A), and the stability of mini-[*PIN* ^+^] isolates (B) is not significantly reduced compared to ΔD3 and Δ3E. In (C) note the significant reduction of prion-containing cells in the cultures expressing the ΔQGB1C2 compared to ΔB1C2 (and lack of prion loss in cells expressing ΔQG). Ability of ΔQGB1C2 to form stable mini-[*PIN* ^+^]s was confirmed in a separate experiment (MK and ID unpublished observations).
(4.02 MB TIF)

Table S1 Constructs for expression of Rnq1 fragments in yeast.
(0.05 MB DOC)

Table S2 Constructs for bacterial expression.
(0.03 MB DOC)

Table S3 Primers used in this study.
(0.07 MB DOC)

Text S1 Mini-*[PIN^+^]*s result from the transmission of the prion state from the pre-existing *[PIN^+^]* prion rather than from *de novo* prion formation.
(0.03 MB DOC)

Protocol S1 Plasmid construction.
(0.04 MB DOC)
