# Supplementary figures and images for: Distinct Type of Transmission Barrier Revealed by Study of Multiple Prion Determinants of Rnq1
Source: PLoS Genet. 2010 Jan 22;6(1):e1000824. doi: 10.1371/journal.pgen.1000824 (PMC2809767; doi:10.1371/journal.pgen.1000824)

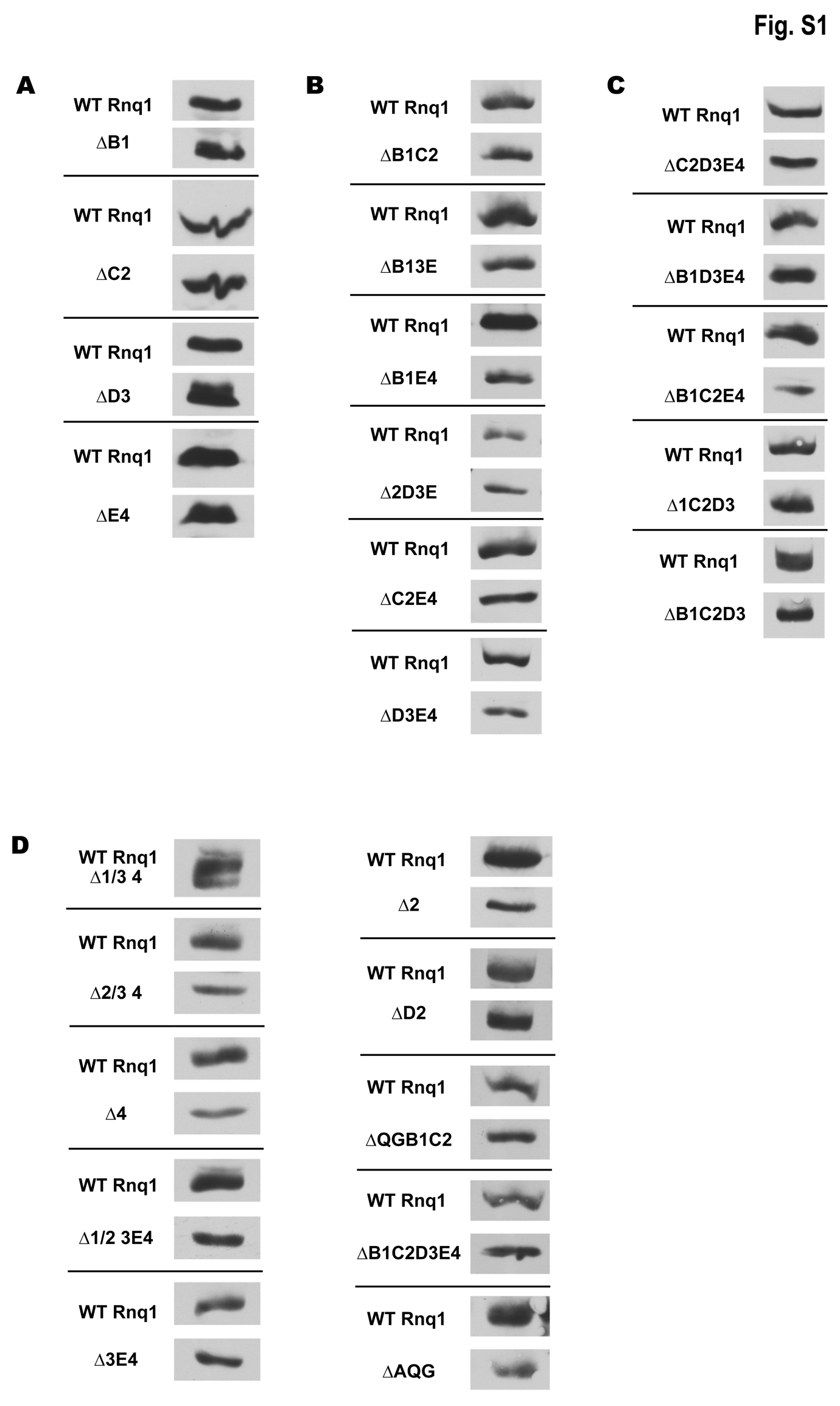

Supplement: Figure S1 — Expression of Rnq1 fragments in yeast. Western blot analysis of the lysates of [PIN+][psi−] rnq1-Δ 74-D694 cells co-expressing Rnq1 and the indicated deletion constructs. Both full-length RNQ1 and its fragments are controlled by the native RNQ1 promoter (see Plasmids in Materials and Methods, and Protocol S1 for plasmid construction). Transformants were maintained on the medium selective for both plasmids, but cultures for protein isolation were grown in YPD. Yeast cell lysates were prepared as described in Liebman et al. [2006] except that pre-clearing at 10,000×g was omitted. Rnq1 was detected with polyclonal antibodies raised against the N-terminal part of the protein ([Lopez et al., 2003]; kindly provided by E. Craig, University of Wisconsin-Madison). Panels show same lanes in the top and bottom parts of the Western blot of the same culture. All Rnq1 fragments ran in accordance with their expected size. At least 2 independent transformants were analyzed for each construct and experiments were repeated 2–5 times. The groups are: deletions of one (A), two (B) and three (C) QN regions with preceding hydrophobic patches used throughout the manuscript; and (D) other constructs used mostly in Figure 5, Figure S2, Figure S8, and Figure S9. (Liebman SW, Bagriantsev SN, Derkatch IL (2006) Biochemical and genetic methods for characterization of [PIN+] prions in yeast. Methods 39: 23–34.) (Lopez N, Aron R, Craig EA (2003) The role of Sis1 on the maintenance of [RNQ+] prion. Mol Biol Cell 14: 1172–1181.) (3.22 MB TIF) [file pgen.1000824.s001.tif]

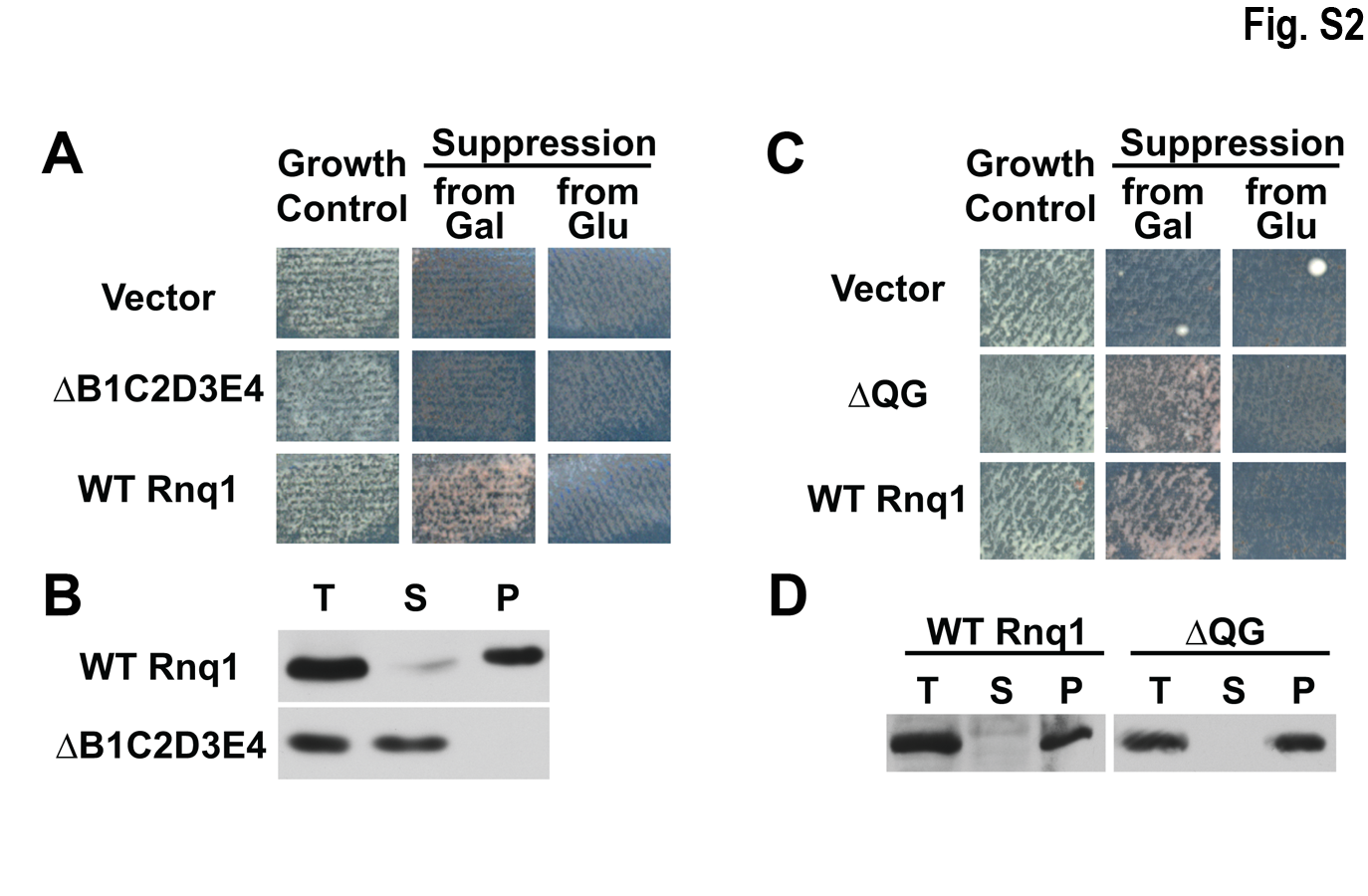

Supplement: Figure S2 — The QN-rich C-terminus is an essential part of the prion domain of Rnq1. N-terminal Rnq1 fragments were previously shown to be unable to join [PIN+] or to transmit the prion state [Vitrenko et al., 2007]. However, in those studies Rnq1 fragments were Gfp-tagged, and large tags sometimes interfere with prion properties [Dagkesamanskaia et al., 1997; Edskes et al., 1999]. We confirm that the QN-rich C-terminus is indispensable for mini-[PIN+]s in our experimental setup, and demonstrate that QG10 is not required for mini-[PIN+] establishment. (A,B) ΔB1C2D3E4, a Rnq1 fragment lacking all QN regions but retaining QG10, does not aggregate in [PIN+] cells and does not carry on the Pin+ phenotype. (A) Cultures expressing ΔB1C2D3E4 become Pin− upon the loss of full-length Rnq1. Plasmid shuffle and [PSI+] induction test were performed as described in Figure 1B legend. The lack of [PSI+] formation was confirmed by fluorescent microscopy using the Sup35NM::Yfp reporter (not shown). (B) There is no evidence of ΔB1C2D3E4 aggregation even in the presence of [PIN+]. Sedimentation analysis of the lysate of [PIN+] cells co-expressing Rnq1 and ΔB1C2D3E4; panels show same lanes in the top and bottom parts of the Western blot of the same culture. Similar data for Δ1C2D3E4 not shown. (C,D) QG10 is not essential for maintaining the prion state of Rnq1: ΔQG is aggregated, and cultures remain Pin+ after elimination of Rnq1. (C) Plasmid shuffle and [PSI+] induction test were performed as described in Figure 1B legend. (D) Sedimentation analysis of cell lysates from cultures expressing indicated fragments after elimination of full-length Rnq1. Similar data for ΔAQG not shown. (Vitrenko YA, Pavon ME, Stone SI, Liebman SW (2007) Propagation of the [PIN+] prion by fragments of Rnq1 fused to GFP. Curr Genet 51: 309–319.) (Dagkesamanskaia AR, Kushnirov VV, Paushkin SV, Ter-Avanesyan MD (1997) Fusion of glutathione S-transferase with the N-terminus of yeast Sup35 protein inhibits its prion-like pro [file pgen.1000824.s002.tif]

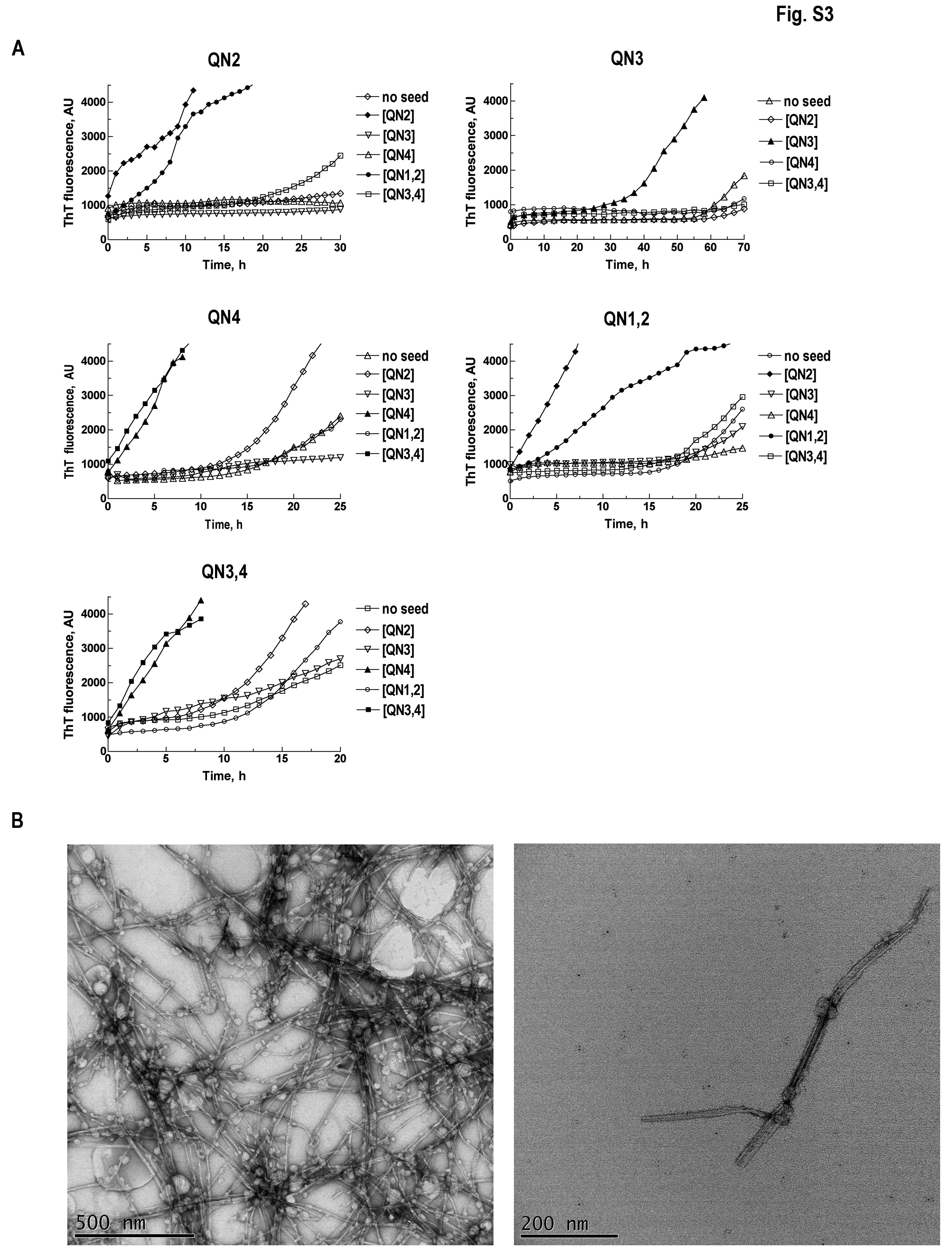

Supplement: Figure S3 — In vitro analysis of aggregation of Rnq1 protein fragments and QN1 peptide. (A) Cross-seeding between Rnq1 protein fragments lacking two or three QN regions. Kinetics of in vitro aggregation was monitored by ThT fluorescence. The soluble protein is indicated above the graphs, and seeds are listed to the right in brackets. “No seed” indicates unseeded polymerization. Proteins used: QN2 (ΔB1D3E4), QN3 (ΔB1C2E4), QN4 (ΔB1C2D3), QN1,2 (ΔD3E4), QN3,4 (ΔB1C2). Concentrations of soluble proteins were in the 60–80 µM range. (B) Transmission electron micrographs of negatively stained QN1 peptide fibers. The 12 aa long QN1 peptide (NSNNNNQQGQNQ; GenScript; 98.8% purity) was pre-treated with 1,1,1,3,3,3,-hexafluoro-2-isopropanol (Sigma) for 24 h at room temperature and lyophilized. The powder was re-suspended in water to a final concentration of 250 µg/ml. The 200 µl reactions were set up in the presence of 5 µM ThT. Samples incubated at 37°C for ∼80 h were shaken for 5 sec every 10 min. Monitoring the aggregation kinetics by ThT fluorescence (see Materials and Methods) revealed a sigmoidal curve with a very short lag phase (not shown). TEM was performed at the NYU School of Medicine Image Core Facility as described in Materials and Methods. Long and very thin (<10 nm in diameter) fibers were frequently laterally associated and had either straight or twisted appearance. (5.34 MB TIF) [file pgen.1000824.s003.tif]

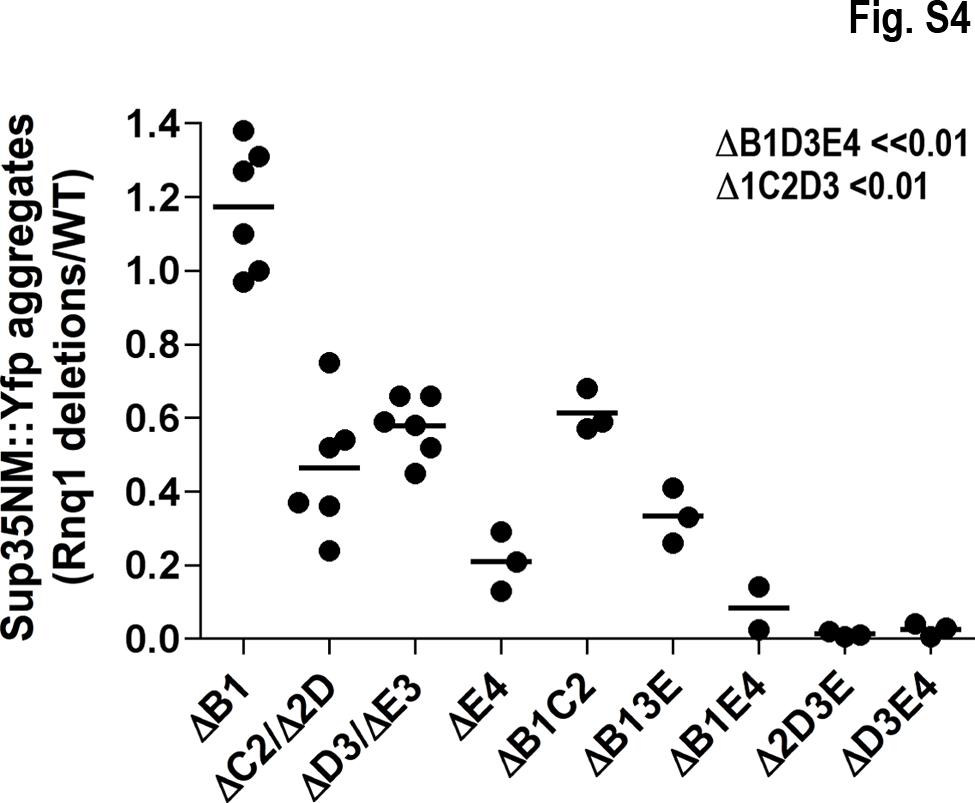

Supplement: Figure S4 — Reduction of the de novo formation of [PSI +] after substitution of deletion constructs for full-length RNQ1. Plasmid shuffle was performed in the [PIN +][psi −] rnq1-Δ 74-D694 strain carrying the pGAL-SUP35NM::YFP [PSI +] inducer as described in Figure 1B legend. [PSI +] was induced by growth on SGal-Leu,His plates for 3 days. Relative levels of [PSI +] induction were calculated by determining the percentage of cells with Sup35NM-Yfp fluorescent aggregates in cultures expressing indicated constructs, and then normalizing it to the percentage of aggregate-containing cells in cultures carrying wild-type [PIN +] only. Each data point represents an independent experiment, in which the percentage of [PSI +] cells determined for three transformants expressing the deletion construct is normalized to the percentage of [PSI +] cells in three transformants expressing wild-type Rnq1 (a total of 500–1,000 cells were analyzed in each case; wild-type [PIN +] cultures carried 30–40% aggregate containing cells). Data for constructs ΔC2 and Δ2D, and ΔD3 and Δ3E were similar and are grouped. Among the constructs lacking only one of the four QN regions, elimination of QN4 had the biggest effect reducing [PSI+] induction ∼5-fold, whereas deleting QN2 or QN3 reduced it ∼2-fold. The effect of double deletions varied depending upon what QN regions were eliminated. Eliminating QN3 in conjunction with a lack of either QN2 or QN4 led to an almost 100-fold drop in [PSI+] induction, whereas deletion of QN1 only mildly increased the effect of eliminating QN3 and QN4 and, surprisingly, had a rescuing effect when deleted together with QN2. Finally, the level of [PSI+] induction was the lowest in cultures expressing the Rnq1 fragments with only one QN region. (2.38 MB TIF) [file pgen.1000824.s004.tif]

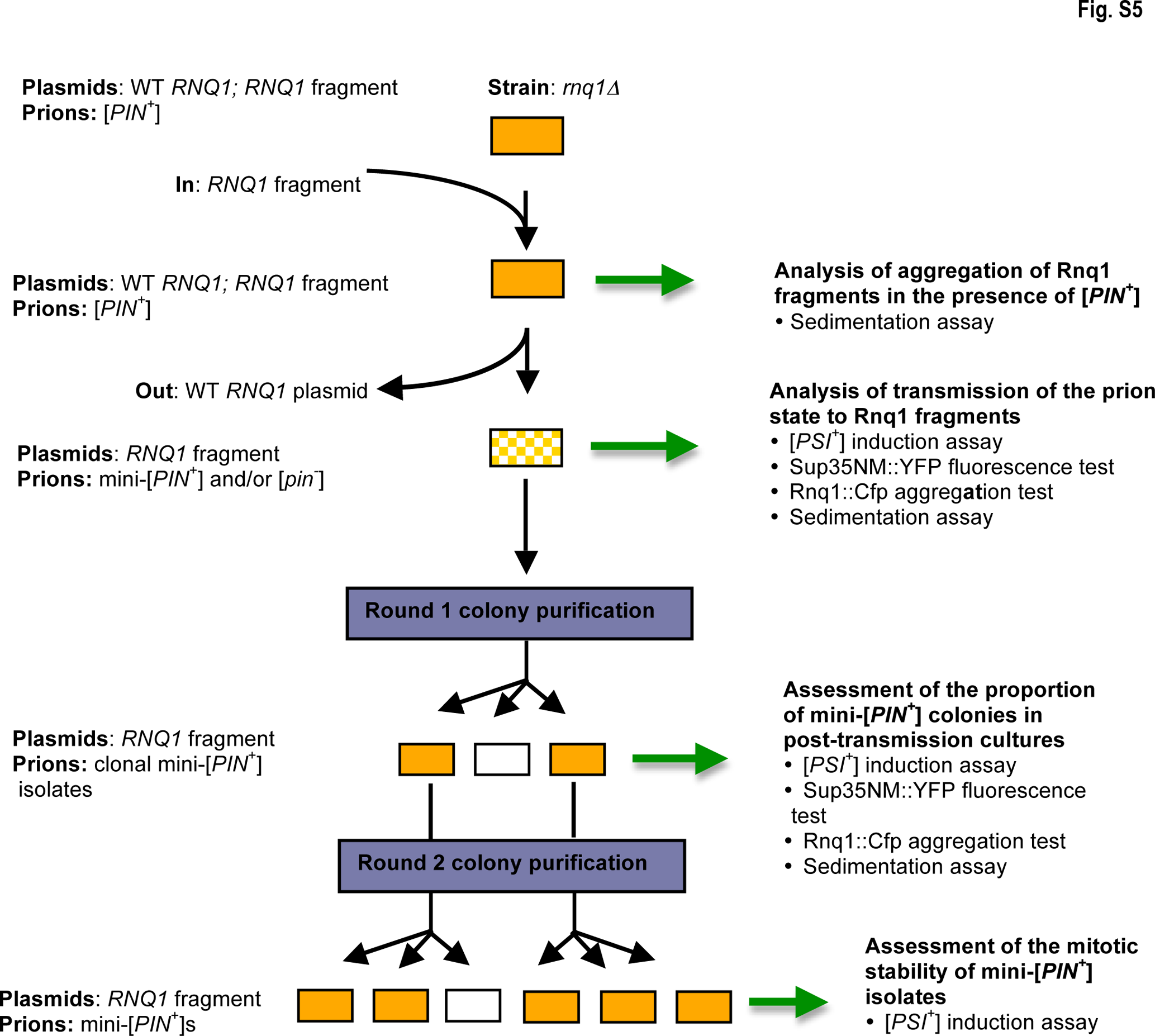

Supplement: Figure S5 — Scheme of the analysis of the transmission of the prion state from [PIN +] to Rnq1 fragments. Experiments are described in the text, Materials and Methods and Legends for Figure 1B and 1C, Figure 4A and 4B. Experiments described in Figure 6 and Figure 7 were performed similarly except the initial strain expressed a Rnq1 fragment and harbored a mini-[PIN +], and the prion state was transmitted to wild-type Rnq1 or to other Rnq1 fragments. (0.57 MB TIF) [file pgen.1000824.s005.tif]

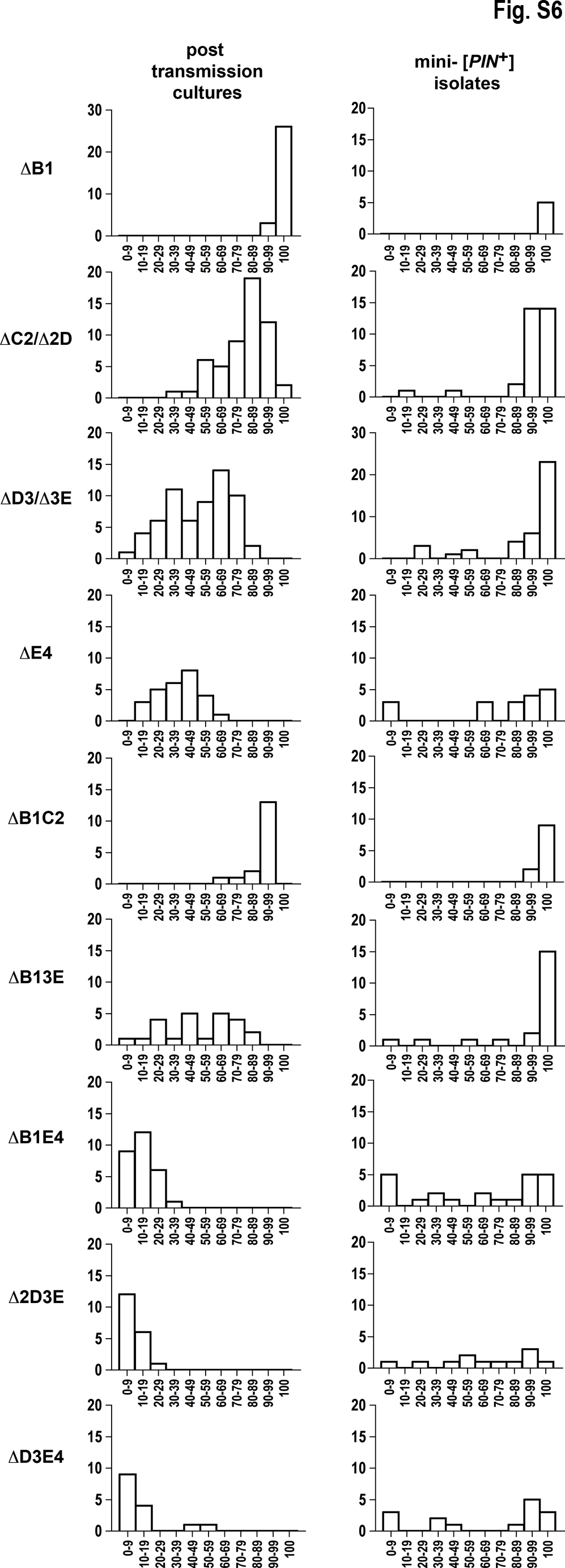

Supplement: Figure S6 — Distributions of percentages of mini-[PIN +] cells in cultures expressing indicated Rnq1 fragments after the loss of wild-type [PIN +]. Analysis of data presented in Figure 4A (Round 1 colony purification) and in Figure 4B (Round 2 colony purification). Horizontal axes show percentage of [PIN +] cells in 10% increments. Vertical axes show frequency of cultures with corresponding percentages of mini-[PIN +] cells. (8.31 MB TIF) [file pgen.1000824.s006.tif]

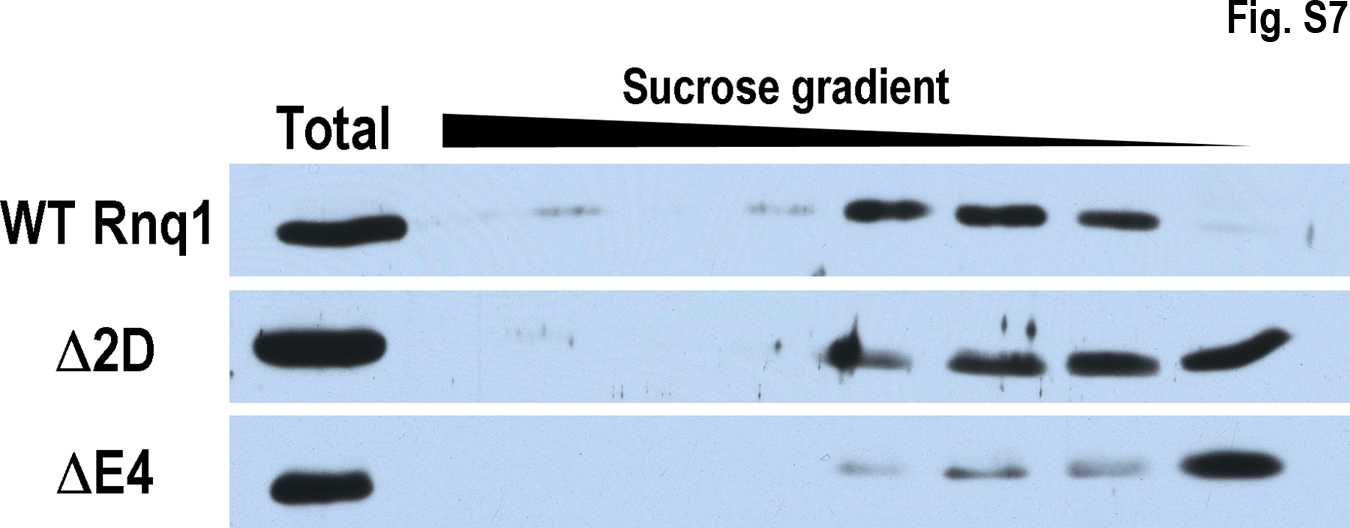

Supplement: Figure S7 — After elimination of full-length Rnq1, Δ2D, and ΔE4 were detected in both soluble and aggregated fractions. For finer analysis of aggregates formed by Rnq1 fragments, 0.5 ml (∼1 mg) of total protein were loaded onto ∼4.5 ml of 15%–40%–60% step sucrose gradient and centrifuged at 160,000×g for 60 min at 4°C (Beckman Optima L-90K centrifuge, SW55Ti rotor). The 0.5 ml fractions were collected from the bottom of the tube, resolved on SDS-PAGE and immunoblotted with anti-Rnq1A. The bottom fraction is not shown. Predominately aggregated wild-type Rnq1 is not detected in the top (soluble) fraction, whereas partially soluble Δ2D and ΔE4 are detected in the top fraction, as well as in the same gradient fractions were WT Rnq1 is present. Consistent with Figure 4A and 4D, ΔE4 lysates have more soluble Rnq1 fragment than Δ2D lysates. (2.17 MB TIF) [file pgen.1000824.s007.tif]

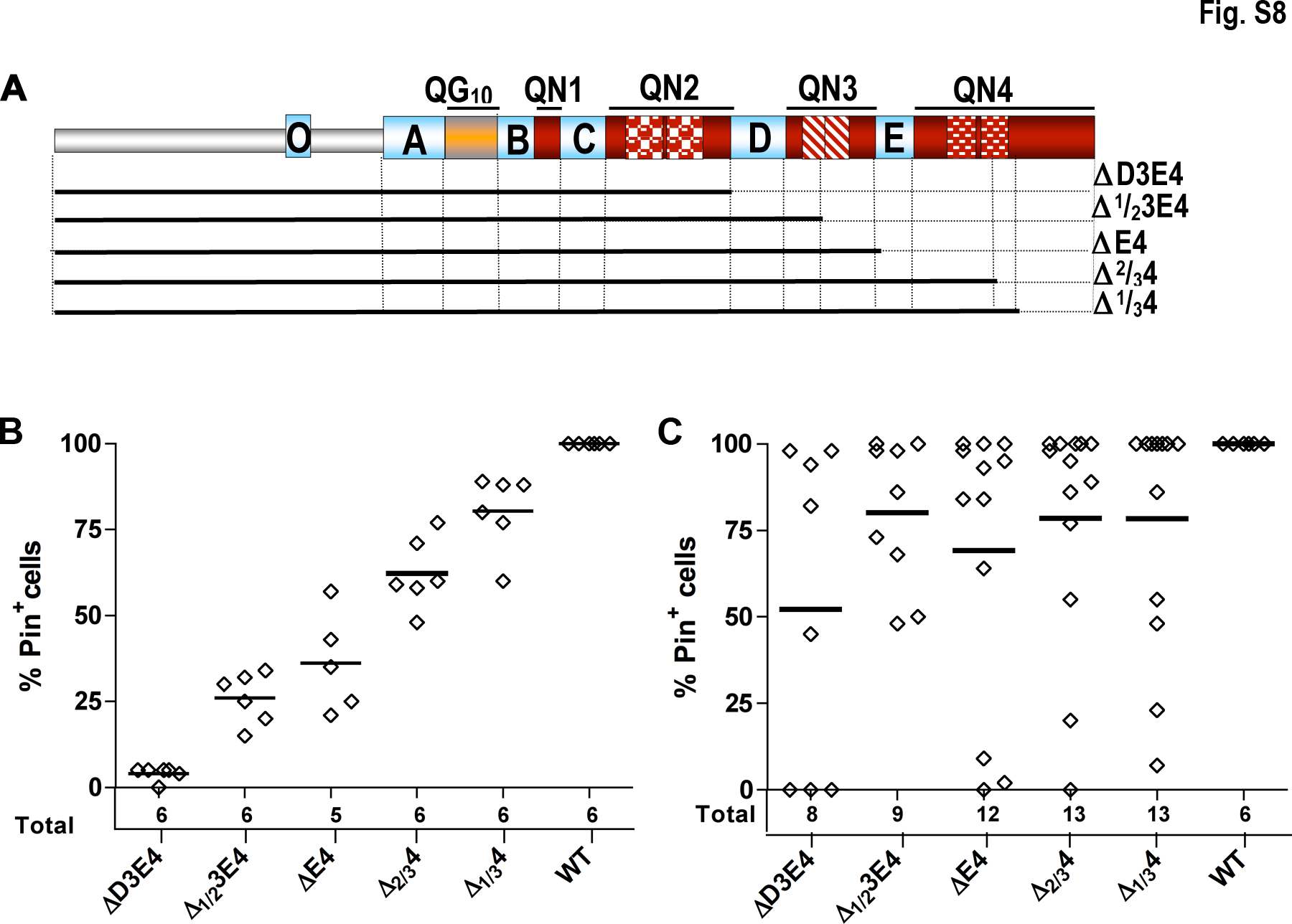

Supplement: Figure S8 — Transmission barrier for conversion of Rnq1 fragments lacking parts of regions QN3 and QN4 into mini-[PIN +]s. (A) Schematic diagram of Rnq1 and deletion constructs used here and in Figure 5A. QN-rich regions are in red; patterned blocks within QN regions indicate oligopeptide repeats; hydrophobic patches are in blue. Lines indicate regions present, and nomenclature refers to deleted regions. Δ1/34 retains the oligopeptide repeat of QN4 but lacks the very C-terminus of Rnq1 that includes a non-QN-rich and a QN-rich stretch. In Δ2/34 only the first oligopeptide of the QN4 region is preserved intact. Δ1/23E4 terminates right after the first oligopeptide of the QN3 repeat. (B) Percentage of mini-[PIN +] cells in cultures bearing indicated deletion constructs after wildtype [PIN +] loss. See Figure 4A legend for full description of the experiment. (C) Analysis of mitotic stability of mini-[PIN +]s formed by the indicated fragments. Data points show percentage of mini-[PIN +] cells in clonal mini-[PIN +] isolates after ∼20 generations of mitotic growth. See Figure 4B legend for full description of the experiement. The total number of independent cutures (B) or mini-[PIN +]s (C) analyzed for each deletion construct is indicated on each graph. Bars indicate averages. In (B) note the gradual decrease in the proportion of mini-[PIN +] cells in cultures with progressive truncations of each QN region. Significant differences between WT Rnq1 and Δ1/34; Δ1/34 and Δ2/34; and Δ2/34 and ΔE4 show that the C-terminal part of QN4, as well as each of the repeated peptides contribute to the transmission barrier (see SEM in Figure 4F). The significant difference between Δ1/23E4 and ΔD3E4 illustrates the contribution of the first of the repeated peptides in QN3. Data in (C) shows that stable mini-[PIN +]s can be obtained after transmission from [PIN +] to any of the fragments. (6.99 MB TIF) [file pgen.1000824.s008.tif]

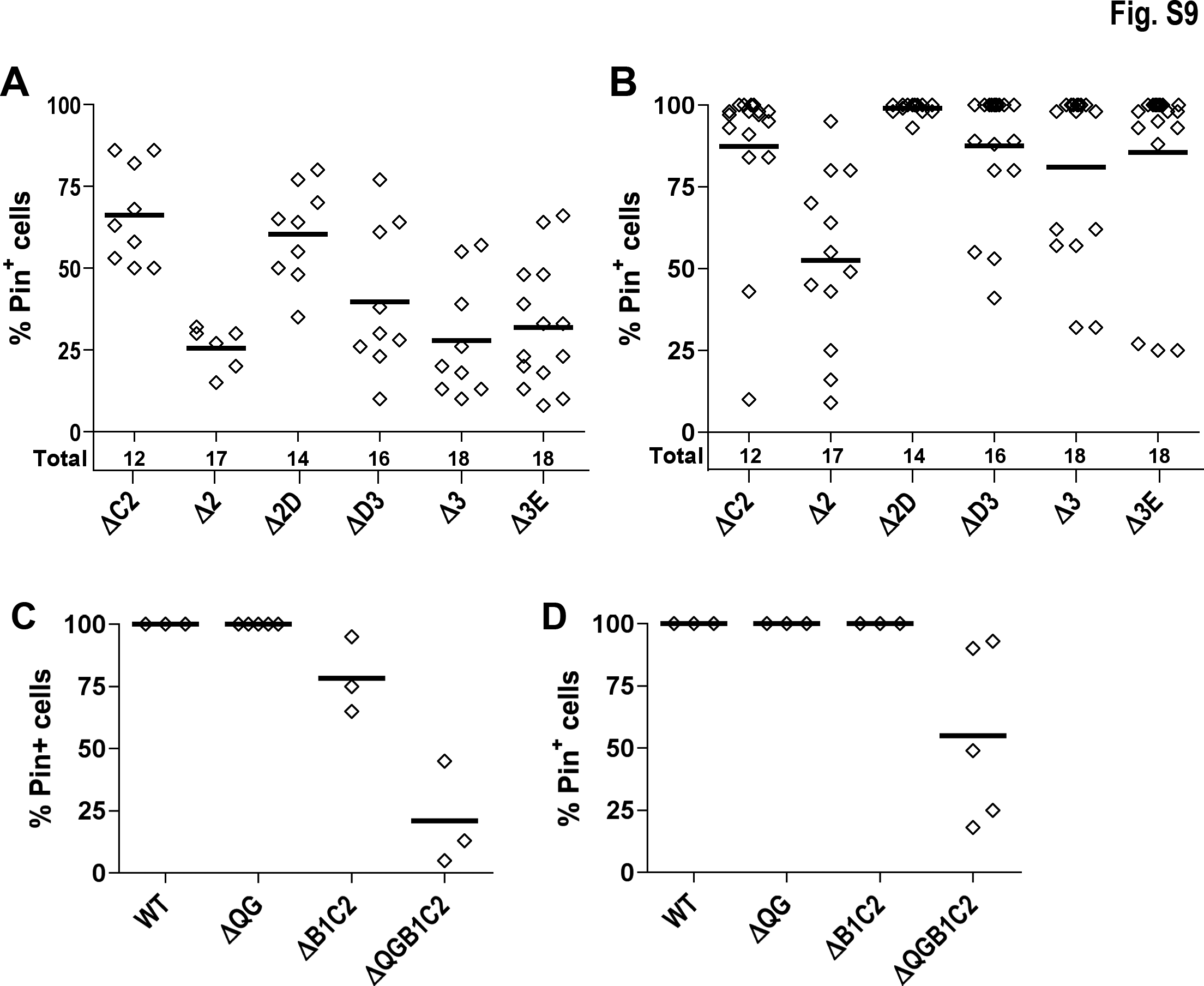

Supplement: Figure S9 — Analysis of the importance of non-QN-rich sequences within the C-terminal part of Rnq1. (A,B) Analysis of the importance of alternating QN regions with hydrophobic patches in the prion domain in Rnq1. (C,D) The QG10 deletion enhances the transmission barrier for the conversion of the Rnq1 fragment lacking QN1 and QN2. (A,C) Percentage of mini-[PIN +] cells in cultures bearing indicated deletion constructs after wildtype [PIN +] loss. (B,D) Analysis of mitotic stability of mini-[PIN +]s formed by the indicated Rnq1 fragments. See Figure 4A and 4B and Figure S8 legends for full description of the experiments. In (A,B) the total number of independent cutures or mini-[PIN +]s analyzed for each deletion construct is indicated on each graph. In (C,D) 3–5 independent cutures or mini-[PIN +]s were analyzed for each deletion construct. In (A), note the significant reduction of prion-containing cells in the cultures expressing the Δ2 Rnq1 fragment (alteration of QN regions and hydrophobic patches is disrupted and hydophobic regions C and D are located next to each other), compared to ΔC2 and Δ2D (alternating pattern is preserved). Analysis of mitotic stability of mini-[PIN +]s formed by these fragments in (B) indicates that the alternating pattern is important for the establishment of stable mini-[PIN +] strains. Yet, although not part of the experiment shown in (B), stable mini-[PIN +] isolates were obtained for Δ2, confirming the ability of this fragment to faithfully maintain the prion state (MK and ID unpublished). For Δ3, the reduction in prion containing cells in post-transmission cultures (A), and the stability of mini-[PIN +] isolates (B) is not significantly reduced compared to ΔD3 and Δ3E. In (C) note the significant reduction of prion-containing cells in the cultures expressing the ΔQGB1C2 compared to ΔB1C2 (and lack of prion loss in cells expressing ΔQG). Ability of ΔQGB1C2 to form stable mini-[PIN +]s was confirmed in a separate experiment (MK and ID unpublished [file pgen.1000824.s009.tif]
