# Supplementary material for: Distinct Type of Transmission Barrier Revealed by Study of Multiple Prion Determinants of Rnq1
Source: PLoS Genet. 2010 Jan 22;6(1):e1000824. doi: 10.1371/journal.pgen.1000824 (PMC2809767; doi:10.1371/journal.pgen.1000824)
Supplement: Table S1 — Constructs for expression of Rnq1 fragments in yeast. (0.05 MB DOC) [file pgen.1000824.s010.doc]

**Table S1.** Constructs for Expression of Rnq1 Fragments in Yeast

| **Construct** | **Primer Pairs for Fragment 1***  **(upstream/ downstream)** | **Primer Pairs for Fragment 2***  **(upstream/downstream** |
| --- | --- | --- |
| ΔB1 | 7/158 | 157/8 |
| ΔC2 | 7/197 | 160/8 |
| Δ2 | 7/159 | 160/8 |
| Δ2D | 7/159 | 161/8 |
| ΔD3 | 7/162 | 163/8 |
| Δ3 | 7/164 | 163/8 |
| Δ3E | 7/164 | 198/8 |
| Δ4 | 7/149 | --- |
| ΔE4 | 7/148 | --- |
| ΔB1C2 | 7/158 | 160/8 |
| ΔB13E** | 7/295 | 294/8 |
| ΔB1E4 | 7/158 | 157/196 |
| Δ2D3E | 7/159 | 198/8 |
| ΔC2E4** | 7/196 | --- |
| ΔD3E4 | 7/145 | --- |
| ΔC2D3E4 | 7/143 | --- |
| ΔB1D3E4** | 7/231 | --- |
| ΔB1C2E4 | 7/158 | 160/196 |
| Δ1C2D3 | 7/156 | 163/8 |
| ΔB1C2D3 | 7/158 | 163/8 |
| ΔB1C2D3E4 | 7/141 | --- |
| Δ1C2D3E4 | 7/142 | --- |
| ΔQG | 7/154 | 155/8 |
| ΔAQ/G | 7/152 | 155/8 |
| ΔQGB1C2 | 7/154 | 160/8 |
| Δ3E4 | 7/146 | --- |
| Δ_1/2_3E4 | 7/147 | --- |
| Δ_2/3_4 | 7/150 | --- |
| Δ_1/3_4 | 7/151 | --- |

* Two PCR fragments were usually used to assemble the constructs carrying *RNQ1* with internal deletions; see Supporting Protocol S1.

** To perform these reactions, *RNQ1* deletion constructs were used as templates; see Supporting Supporting Protocol S1.
