# Supplementary material for: Distinct Type of Transmission Barrier Revealed by Study of Multiple Prion Determinants of Rnq1
Source: PLoS Genet. 2010 Jan 22;6(1):e1000824. doi: 10.1371/journal.pgen.1000824 (PMC2809767; doi:10.1371/journal.pgen.1000824)
Supplement: Table S2 — Constructs for bacterial expression. (0.03 MB DOC) [file pgen.1000824.s011.doc]

**Table S2.** Constructs for Bacterial Expression

| **Bacterial Expression**  **Construct** | **Corresponding Yeast**  **Construct** | **Primers** | |
| --- | --- | --- | --- |
|  |  | **Upstream** | **Downstream** |
| QN1 | ΔC2D3E4 | 300 | 301 |
| QN2 | ΔB1D3E4 | 300 | 302 |
| QN3 | ΔB1C2E4 | 300 | 303 |
| QN4 | ΔB1C2D3 | 300 | 304 |
| QN1,2 | ΔD3E4 | 300 | 302 |
| QN3,4 | ΔB1C2 | 300 | 304 |
