# Supplementary material for: Distinct Type of Transmission Barrier Revealed by Study of Multiple Prion Determinants of Rnq1
Source: PLoS Genet. 2010 Jan 22;6(1):e1000824. doi: 10.1371/journal.pgen.1000824 (PMC2809767; doi:10.1371/journal.pgen.1000824)
Supplement: Table S3 — Primers used in this study. (0.07 MB DOC) [file pgen.1000824.s012.doc]

**Table S3.** Primers Used in This Study

| **Primer Number** | **Primer Sequence (5’ 🡪 3’)** |
| --- | --- |
| 3 | GCGGGATCCAGATCTTTGCTATACGTTTGT |
| 7 | GGCGGATCCATGGATACGGATAAGTTAATCTC |
| 8 | AGGCCGCGGGTAGCGGTTCTGGTTGC |
| 19 | CCGGAATTCCATGGTATTTCAAACGCA |
| 29 | TCCCCGCGGTGAACGATGATTCAGTTCG |
| 30 | TCCGAGCTCAAAATTGTCTTGCAGCCCA |
| 31 | TCCCCGCGGCAAGACAATTTTGTTGTCA |
| 32 | CGTCCGAGCTCGTCTGCATTATTGTTGCTA |
| 141 | AGGCCGCGGTCAAGAACCTTGACCTTGACC |
| 142 | AGGCCGCGGTCAGGAATTCATGAAAGATGAAG |
| 143 | AGGCCGCGGTCAGGAGGAACCACCGGAG |
| 145 | AGGCCGCGGTCAACCACCAGATTGTCCC |
| 146 | AGGCCGCGGTCAACCTAAGTAAGATTGAGCCATG |
| 147 | AGGCCGCGGTCAGTTGTTTTGGCCTTGTTG |
| 148 | AGGCCGCGGTCATGAACTGGAGTGGCCTTG |
| 149 | AGGCCGCGGTCAGCCCAGGTAGGAACTTG |
| 150 | AGGCCGCGGTCAATTGGATTGCTGTTGACC |
| 151 | AGGCCGCGGTCACTGTCCATTGGAGTTCTG |
| 152 | TTTTCCGGATTGGCTCCCGCCGCC |
| 154 | TTTTCCGGAATTGTTACCTGACTTAAAG |
| 155 | TTTTCCGGATCTTTTACTGCTTTGGC |
| 156 | TTTTCCGGAATTCATGAAAGATGAAGC |
| 157 | TTTTCCGGAGGTTCCTCCTTTGGAG |
| 158 | TTTTCCGGAACCTTGACCTTGACC |
| 159 | TTTTCCGGAATGCATAAAAGAGCTTGC |
| 160 | TTTTCCGGAGGTGCTTTTTCCTCATTG |
| 161 | TTTTCCGGAGGTGGACAAACTCAATC |
| 162 | TTTTCCGGATTGTCCCTGTTGTTG |
| 163 | TTTTCCGGACACTCCAGTTCATTCTC |
| 164 | TTTTCCGGATTGAGTTTGTCCACC |
| 196 | AGGCCGCGGTGAACTGGAGTGGCCTTGTTG |
| 197 | TTTTCCGGAGCTTTGATTTTGACCTTG |
| 198 | TTTTCCGGAAATAACTCCAATTCAAATTC |
| 231 | AGGCCGCGGACCACCAGATTGTCCC |
| 294 | TTTTGCCTCCATGGCTCAATCTTAC |
| 295 | TTTTGCCATGGAGGCCAATGAGG |
| 300 | GGAATTCCATATGGATACGGATAAGTTAATC |
| 301 | GCGGGCCCTTATTGATTTTGACCTTGCTGAT |
| 302 | GCGGGCCCTTATTGTCCCTGTTGTTGTTGGT |
| 303 | GCGGGCCCTTATTGTTGCTGCTGCTGACCCT |
| 304 | GCGGGCCCTTAGTAGCGGTTCTGGTTGCCGT |

Restriction sites underlined: *Bam*HI (GGATCC), *Eco*RI (GAATTC), *Sac*I (GAGCTC), *Sac*II (CCGCGG), *Bsp*EI (TCCGGA), *Bgl*I (GCCNNNNNGGC), *Apa*I (GGGCCC), *Nde*I (CATATG).
