# Supplementary material for: Distinct Type of Transmission Barrier Revealed by Study of Multiple Prion Determinants of Rnq1
Source: PLoS Genet. 2010 Jan 22;6(1):e1000824. doi: 10.1371/journal.pgen.1000824 (PMC2809767; doi:10.1371/journal.pgen.1000824)
Supplement: Text S1 — Mini-[PIN+]s result from the transmission of the prion state from the pre-existing [PIN+] prion rather than from de novo prion formation. (0.03 MB DOC) [file pgen.1000824.s013.doc]

**Text S1. Mini-[*PIN+*]s result from the transmission of the prion state from the pre-existing [*PIN+*] prion rather than from *de novo* prion formation**

To address the concern that mini-[*PIN+*]s could form *de novo* at high frequency if respective Rnq1 fragments were overexpressed, deletion fragments were expressed from single copy plasmids under the control of the native *RNQ1* promoter. Western blot analyses showed similar levels of Rnq1 in lysates of 74-D694 and *rnq1*-Δ 74-D694 carrying a *RNQ1* plasmid (not shown), as well as lack of over-accumulation of Rnq1 fragments compared to full-length Rnq1, when both were expressed from plasmids (Figure S1). In the latter experiments Rnq1 was detected with the Rnq1A antibody (kindly provided by E. Craig), which was raised against the protein encompassing only the N-terminal region of Rnq1 [Lopez et al., 2003] and is expected to recognize all Rnq1 fragments with similar efficiency.

To exclude the possibility that some Rnq1 fragments are prone to take on the prion conformation even when expressed at physiological levels, transmission experiments were repeated in the [*psi−*][*pin−*] *rnq1*-Δ 74-D694. We found no evidence that any of the constructs was prone to take the prion conformation in [*pin-*] cells (not shown). Analogously, comparison of extracts from [*PIN+*] and [*pin-*] 74-D694 cells co-expressing full-length Rnq1 and deletion constructs confirmed that Rnq1 fragments became insoluble only in the presence of pre-existing [*PIN+*], but remained soluble in the absence of the template (not shown).

1. Lopez N, Aron R, Craig EA (2003) The role of Sis1 on the maintenance of [*RNQ­­+­*] prion. Mol Biol Cell 14: 1172-1181.
