# Supplementary material for: Distinct Type of Transmission Barrier Revealed by Study of Multiple Prion Determinants of Rnq1
Source: PLoS Genet. 2010 Jan 22;6(1):e1000824. doi: 10.1371/journal.pgen.1000824 (PMC2809767; doi:10.1371/journal.pgen.1000824)
Supplement: Protocol S1 — Plasmid construction. (0.04 MB DOC) [file pgen.1000824.s014.doc]

**Protocol S1**

**Plasmid Construction**

Plasmids for expression of *RNQ1* and its deletion alleles in yeast were constructed on the backbone of pRS416 (*URA3)* or pRS415 (*LEU2)* *CEN* vectors [Sikorski and Hieter, 1989]. pRS416 and pRS415 were also used as empty vector controls. The *RNQ1* promoter amplified with primers #19 and #3 (see Table S3 for primer sequences) was inserted into the *Eco*RI and *Bam*HI sites. The *RNQ1* terminator amplified with primers #29 and #30 was cloned into the *Sac*II and *Sac*I sites. *RNQ1* alleles were cloned as *Bam*HI - *Sac*II fragments between the promoter and terminator. Wild type *RNQ1* ORF was amplified with primers #7 and #8; the resulting plasmids are pID127 (*URA3)* and pID129 (*LEU2*), respectively.

*RNQ1* fragments for C-terminal deletion constructs were amplified using the same upstream primer, #7, and a series of downstream primers where a stop codon preceded the *Sac*II site. Combinations of primers used to obtain deletion constructs are listed in Table S1. To obtain internal deletions, two separate *RNQ1* fragments were PCR-amplified for each construct: the first corresponded to the portion of *RNQ1* ORF preceding the deletion, and the second - to the remainder of the gene past the deletion. The downstream primer for the first fragment and the upstream primer for the second fragment encompassed in-frame *Bsp*EI sites. The two PCR fragments were digested with *Bsp*EI and ligated. The ligation products where the two fragments were fused were re-amplified with the upstream primer for the first fragment (#7) and the downstream primer for second fragment (#8) and cloned into the *Bam*HI and *Sac*II sites of the vector. Ligation of *Bsp*EI-digested fragments produced *RNQ1* ORFs with internal deletions and a sequence coding for SG at the junction. The presence of S and G residues at the borders of QG_10_ and QN-rich regions made it possible to create precise seamless deletions of all sequence determinants in the C-terminal part of Rnq1. The only change introduced was at the DNA level where S and G codons were recoded to TCC and GGA, respectively (according to *S. cerevisiae* codon usage table at http://www.kazusa.or.jp/codon/index.html these changes were unlikely to affect protein synthesis). ΔB1C2E4 and ΔB1E4 were constructed similarly except using the #196 downstream primer for the second PCR fragment. ΔC2E4 and ΔB1D3E4 were made like other C-terminal deletions, but from ΔC2 and ΔB1E4, respectively. To obtain ΔB13E, *RNQ1* was amplified from ΔB1and Δ3E constructs, PCR products were digested with *Bgl*I (the site is located in hydrophobic region D) and fused together; the double-deletion ligation product was re-amplified and cloned into the *Bam*HI and *Sac*II sites of the vector.

Plasmids for bacterial expression were obtained by amplifying *RNQ1* fragments from corresponding yeast constructs (see Table S2 for construct names and primer numbers), and inserting them as *Apa*I - *Nde*I restriction fragments into pJC45 [Clos and Brandau, 1994] to yield N-terminally 10xHIS-tagged proteins. Similarly constructed plasmid for expression of full-length Rnq1 was kindly provided by S. Liebman (University of Illinois at Chicago; [Vitrenko et al., 2007]).

For inducing and quantifying the *de novo* formation of [*PSI^+^*], the *CEN HIS3*-marked pRS413-based pGAL-SUP35NM::YFP (pID106) was constructed by substituting the *GAL1* promoter (the *Bam*HI – *Eco*RI fragment from pRS316-GAL1; [Liu et al., 1992]) for the *CUP1* promoter in pID56 (pNM-YFP in [Derkatch et al., 2004]). The *CEN URA3*-marked pCUP-RNQ::CFP (pID104) is identical to previously described *RNQ1*-*CFP* fusions [Derkatch et al., 2001; 2004] but carries wild type *RNQ1* ORF amplified with primers #7 and #8.

1. Sikorski RS, Hieter P (1989) A system of shuttle vectors and yeast host strains designed for efficient manipulation of DNA in *Saccharomyces cerevisiae*. Genetics 122: 19-27.

2. Clos J, Brandau S (1994) pJC20 and pJC40 - two high-copy-number vectors for T7 RNA polymerase-dependent expression of recombinant genes in *Escherichia coli*. Protein Expr Purif 5: 133-137.

3. Vitrenko YA, Gracheva EO, Richmond JE, Liebman SW (2007) Visualization of aggregation of the Rnq1 prion domain and cross-seeding interactions with Sup35NM. J Biol Chem 282: 1779-1787.

4. Liu H, Krizek J, Bretscher A (1992) Construction of *GAL1*-regulated yeast cDNA expression library and its application to the identification of genes whose overexpression causes lethality in yeast. Genetics 132: 665-673.

5. Derkatch IL, Uptain SM, Outeiro TF, Krishnan R, Lindquist SL, Liebman SW (2004) Effects of Q/N, polyQ and non-polyQ amyloids on the de novo formation of the [*PSI^+^*] prion in yeast and aggregation of Sup35 in vitro. Proc Natl Acad Sci USA 101: 12934-12939.

6. Derkatch IL, Bradley ME, Hong J, Liebman SW (2001) Prions affect the appearance of other prions: the story of [*PIN^+^*]. Cell 106: 171-182.
